# Bridge RNAs direct programmable recombination of target and donor DNA

**DOI:** 10.1038/s41586-024-07552-4

**Published:** 2024-06-26

**Authors:** Matthew G. Durrant, Nicholas T. Perry, James J. Pai, Aditya R. Jangid, Januka S. Athukoralage, Masahiro Hiraizumi, John P. McSpedon, April Pawluk, Hiroshi Nishimasu, Silvana Konermann, Patrick D. Hsu

**Affiliations:** 1 Arc Institute, Palo Alto, CA USA; 2 Department of Bioengineering, University of California, Berkeley, Berkeley, CA USA; 3 University of California, Berkeley–University of California, San Francisco Graduate Program in Bioengineering, Berkeley, CA USA; 4 Department of Chemistry and Biotechnology, Graduate School of Engineering, University of Tokyo, Tokyo, Japan; 5 Structural Biology Division, Research Center for Advanced Science and Technology, University of Tokyo, Tokyo, Japan; 6 Department of Biological Sciences, Graduate School of Science, University of Tokyo, Tokyo, Japan; 7 Inamori Research Institute for Science, Kyoto, Japan; 8 Japan Science and Technology Agency, Core Research for Evolutional Science and Technology, Saitama, Japan; 9 Department of Biochemistry, Stanford University School of Medicine, Stanford, CA USA; 10 Center for Computational Biology, University of California, Berkeley, Berkeley, CA USA

**Keywords:** Microbial genetics, DNA recombination, Transposition, Non-coding RNAs

## Abstract

Genomic rearrangements, encompassing mutational changes in the genome such as insertions, deletions or inversions, are essential for genetic diversity. These rearrangements are typically orchestrated by enzymes that are involved in fundamental DNA repair processes, such as homologous recombination, or in the transposition of foreign genetic material by viruses and mobile genetic elements^1,2^. Here we report that IS110 insertion sequences, a family of minimal and autonomous mobile genetic elements, express a structured non-coding RNA that binds specifically to their encoded recombinase. This bridge RNA contains two internal loops encoding nucleotide stretches that base-pair with the target DNA and the donor DNA, which is the IS110 element itself. We demonstrate that the target-binding and donor-binding loops can be independently reprogrammed to direct sequence-specific recombination between two DNA molecules. This modularity enables the insertion of DNA into genomic target sites, as well as programmable DNA excision and inversion. The IS110 bridge recombination system expands the diversity of nucleic-acid-guided systems beyond CRISPR and RNA interference, offering a unified mechanism for the three fundamental DNA rearrangements—insertion, excision and inversion—that are required for genome design.

## Abstract (web summary)

A bispecific non-coding RNA expressed by the IS110 family of mobile genetic elements forms the basis of a programmable genome-editing system that enables the insertion, excision or inversion of specific target DNA sequences.

## Main

Evolution has dedicated a vast number of enzymes to the task of rearranging and diversifying the genome. This process enables the emergence and functional specialization of new genes, the development of immunity^3^ and the opportunistic spread of viruses and mobile genetic elements (MGEs)^1,2^. MGEs are abundant throughout all domains of life and often mobilize through a transposase, integrase, homing endonuclease or recombinase. These enzymes typically recognize DNA through protein–DNA contacts and can be broadly classified by their target sequence specificity, which ranges from site-specific (for example, Cre and Bxb1 recombinases)^4,5^ to semi-random (for example, Tn5 and PiggyBac transposases)^6,7^.

Insertion sequence (IS) elements are among the most minimal autonomous MGEs, and are found abundantly across bacteria and archaea. Many characterized IS elements use a self-encoded transposase that recognizes terminal inverted repeats (TIRs) through protein–DNA interactions^8^. IS elements have been categorized into approximately 28 families on the basis of their homology, architecture and transposition mechanisms, but they can be broadly grouped by the conserved catalytic residues of their encoded transposases. These include DDE, DEDD and HUH transposases, and, less frequently, serine or tyrosine transposases^8^.

IS110 family elements are cut-and-paste MGEs that scarlessly excise themselves from the genome and generate a circular form as part of their transposition mechanism^9,10^. Given what is known about this mechanism and life cycle, IS110 transposases are more accurately described as recombinases. Although circular intermediates are found in other IS families, IS110 is the only family that uses a DEDD catalytic motif in its recombinase. The N-terminal DEDD domains of IS110 recombinases share homology with RuvC Holliday junction resolvases, suggesting that they have a unique mechanism of action compared with other IS elements. IS110 elements typically lack TIRs and appear to integrate in a sequence-specific manner, often targeting repetitive elements in microbial genomes^11^. Although the mechanism of DNA recognition and recombination for IS110 elements remains unclear, previous studies have suggested that the non-coding ends of the element flanking the recombinase ORF regulate recombinase expression^12,13^.

Here we show that the IS110 circular form drives the expression of a non-coding RNA (ncRNA) with two distinct binding loops that separately recognize the IS110 DNA donor and its genomic insertion target site. By bridging the donor and target DNA molecules through direct base-pairing interactions, the bispecific bridge RNA facilitates DNA recombination by the IS110 recombinase. Each binding loop of the bridge RNA can be independently reprogrammed to bind and recombine diverse DNA sequences. We further show that this modularity enables a generalizable mechanism for DNA rearrangement through sequence-specific insertion, inversion and excision.

## IS621 recombinase binds to a ncRNA

**Fig. 1:**
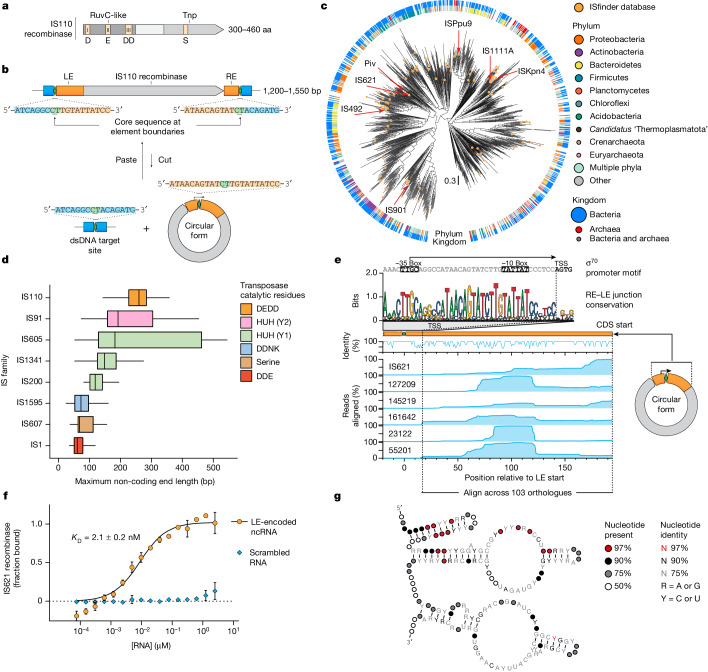
IS110 mobile genetic elements express a ncRNA that is bound by its encoded recombinase. **a**, Schematic representation of the IS110 recombinase protein sequence. **b**, Schematic representation of the structure and life cycle of an IS110 element. Core sequences are depicted as green diamonds, the genomic target site is shown in blue and the non-coding ends are orange. Sequences are from IS621. **c**, A midpoint-rooted phylogenetic tree constructed from 1,054 IS110 recombinase sequences. **d**, Distribution of non-coding end lengths across eight IS families. The maximum of the LE and RE lengths is plotted for each family. Box plots show median (centre line), interquartile range (IQR) (box edges) and 1.5 × IQR (whiskers). Outliers not shown. *n* = 268 for IS110; *n* = 18–184 for other families (Extended Data Fig. 2). **e**, Small RNA-seq coverage plot of the concatenated non-coding ends of IS621 and five related orthologues expressed from their endogenous promoter in *E. coli*. Top, sequence logo of the conservation of the σ^70^ promoter motif. TSS, transcription start site. **f**, MST of a fluorescently labelled IS621 recombinase with either WT or scrambled ncRNA to measure the equilibrium dissociation constant (*K*_D_). Mean ± s.d. of three technical replicates. **g**, Consensus secondary structure of ncRNAs constructed from 103 IS110 LE sequences.

**Extended Data Fig. 1:**
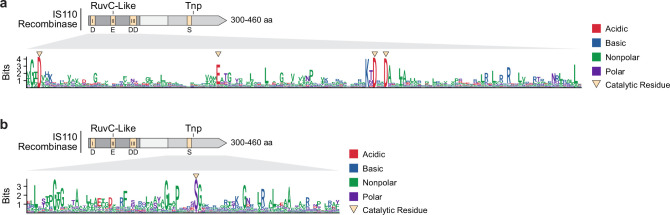
Conserved residues in the RuvC-like and Tnp domains of IS110. **a**, Sequence logo of 213,171 aligned RuvC-like domains identified in IS110 protein sequences. The RuvC-like and Tnp domains shown here were identified using hmmsearch and Pfam models DEDD_Tnp_IS110 (PF01548.19) and Transposase_20 (PF02371.18), respectively. RuvC-like domains were aligned using hmmalign, and these alignments were visualized to identify conserved residues. The conserved residues of the characteristic DEDD motif are highlighted with an arrowhead. The y-axis indicates entropy at each position as measured in bits, with log_2_20 ≈ 4.32 bits being maximally conserved. **b**, Sequence logo of 208,634 aligned Tnp domains identified in IS110 protein sequences. The Tnp domains were identified, extracted, and analysed using the same procedure as for the RuvC-like domain. A highly conserved serine residue is highlighted with an arrowhead. The y-axis indicates entropy at each position as measured in bits, with log_2_20 ≈ 4.32 bits being maximally conserved.

IS110 elements encode recombinases that are around 300–460 amino acids (aa) in length and have an N-terminal DEDD RuvC-like domain (Pfam ID: PF01548) and a C-terminal domain with a highly conserved serine residue^8,14^ (Pfam ID: PF02371) (Fig. 1a and Extended Data Fig. 1a,b). They use this recombinase to scarlessly excise out of their genomic context, yielding a double-stranded DNA (dsDNA) circular form that is inserted into specific genomic target sequences such as repetitive extragenic palindromic (REP) elements^9,12,15,16^ (Fig. 1b and Supplementary Table 1). Recombination of the circular form and the target centres around a short core sequence, and the intervening sequences between the cores and the recombinase coding sequence (CDS) are defined as the left (LE) and right (RE) non-coding ends. IS110 recombinases are highly diverse and widespread in prokaryotes, but only a small subset have been catalogued by curated databases or functionally characterized (Fig. 1c).

**Extended Data Fig. 2:**
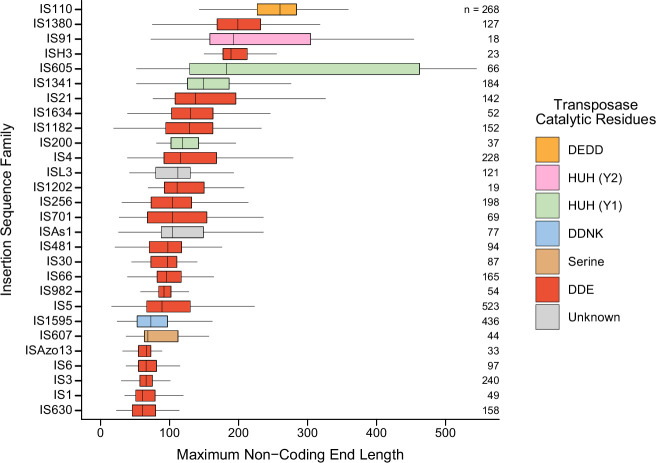
Maximum non-coding end length distribution of 28 IS families. Distribution of non-coding end lengths across different IS families. Box plot depicting the distribution of non-coding end lengths across IS families, calculated using maximum RE–LE length of 90% identity clusters. Box plots show median (centre line), IQR (box edges) and 1.5 × IQR (whiskers). Outliers not shown.

We found that IS110s have the longest median non-coding end lengths, with a relatively narrow distribution, compared with other IS families (Fig. 1d, Extended Data Fig. 2). Upon excision, the circular form of the element reconstitutes a promoter across the core sequence of the concatenated RE–LE far upstream of the recombinase CDS^12,13^ (Fig. 1b), which suggests that a ncRNA could be expressed from this region. Previous reports have shown that the non-coding ends of IS200 and IS605 family elements are transcribed into RNAs that resemble CRISPR RNAs to guide endonuclease activity^17,18^, and small RNAs have been thought to modulate recombinase expression for the IS110 family member ISPpu9 (ref. ^19^).

To investigate the potential presence of an IS110-encoded ncRNA, we focused on the IS110 family member IS621, which is native to some strains of *Escherichia coli*, and five closely related orthologues (Supplementary Table 2). Small RNA sequencing (RNA-seq) of *E. coli* containing a plasmid that encodes the concatenated RE–LE sequences of the predicted circular forms revealed a continuous peak spanning around 177 bp of the LE, starting from the predicted endogenous σ^70^-like promoter (Fig. 1e).

Next, we measured the affinity of an in-vitro-transcribed 177-nucleotide (nt) ncRNA from IS621 and its purified cognate recombinase using microscale thermophoresis (MST). We found that the IS621 recombinase binds to the LE-encoded ncRNA, but not to a scrambled 177-nt RNA control, with high affinity (dissociation constant (*K*_D_) = 2.1 ± 0.2 nM) (Fig. 1f). Our data indicate that IS110 element excision reconstitutes a promoter to drive the expression of a ncRNA that specifically binds to its recombinase enzyme, suggesting that the ncRNA might have a role in recombination.

## ncRNA covaries with target and donor DNA

**Extended Data Fig. 3:**
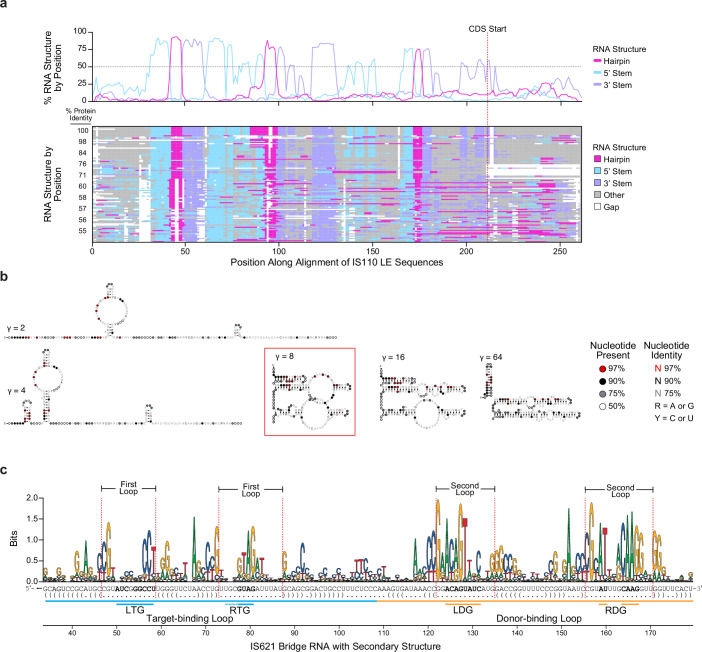
Secondary structure alignment of IS621 non-coding ends and consensus secondary structure prediction. **a**, Secondary RNA structure alignment of the LE of 103 orthologues of IS621. Secondary RNA structures of the LE of 103 orthologues are predicted and aligned by cluster identity. The percentage of each position corresponding to a 5′ stem, hairpin, or 3′ stem are plotted with a dotted line indicating structures that are conserved in over 50% of sequences. For LE sequences shown along the y-axis, the similarity of their cognate proteins relative to the IS621 recombinase is indicated. This type of visualization was often used throughout the study to determine the presence or absence of a structured ncRNA sequence in the flanks of IS110 recombinase ORFs. **b**, RNA structures predicted from the LE sequence alignment in **a**. RNA structures were predicted using ConsAliFold, which uses a parameter γ to control the prediction balance between positive values (or sequence alignment column base-pairings) and negative values (or unpaired sequence alignment columns). Higher values of γ result in more predicted base-pairing. Showing structures resulting from γ = 2, γ = 4, γ = 8, γ = 16, and γ = 64. The value γ = 8 was used for the initial IS621 ncRNA model in this study. **c**, Nucleotide conservation across the predicted ncRNA. 2,715 ncRNA orthologue sequences were identified using an iterative search with the original IS621 model, and then aligned with cmalign. The x-axis indicates conservation of nucleotides as measured in bits, quantifying entropy. Highlighting the regions within the prominent internal loops with dotted red lines, with dot-bracket RNA secondary structure notation along the x-axis. The first loop has low sequence conservation (average information content = 0.48 ± 0.09), while the second one is much more conserved (average information content = 0.93 ± 0.11). Sequence features of the bridge RNA are highlighted for clarity.

We evaluated the ncRNA consensus secondary structure across 103 diverse orthologues, and revealed a 5′ stem-loop followed by two additional stem-loops with prominent internal loops (Fig. 1g and Extended Data Fig. 3a,b). The first internal loop has relatively low sequence conservation across orthologues, whereas the second is much more conserved (Extended Data Fig. 3c).

**Fig. 2:**
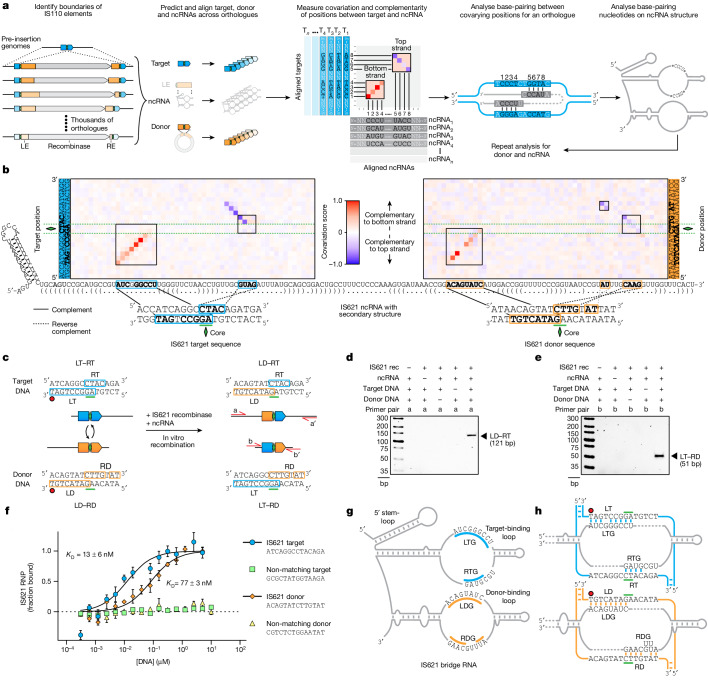
Identification of IS621 bridge RNA binding loops with sequence-specific recognition of target and donor DNA. **a**, Schematic of the computational approach to assess the base-pairing potential between the IS110 ncRNA and its cognate genomic target site or donor sequence. Covariation analysis between target–ncRNA or donor–ncRNA pairs yields a matrix in which diagonal stretches of red signal indicate ncRNA complementarity to the bottom strand of the DNA and blue stretches indicate complementarity to the top strand. **b**, Nucleotide covariation and base-pairing potential between the ncRNA and the target (left) and donor (right) sequences across 5,511 ncRNA–target pairs and 2,201 ncRNA–donor pairs. The IS621 ncRNA sequence is shown across the *x* axis, along with dot-bracket notation predictions of the secondary structure. Covariation scores are coloured according to strand complementarity, with −1 (blue) representing high covariation and a bias toward top-strand base-pairing, and 1 (red) representing high covariation and a bias toward bottom-strand base-pairing. Regions of notable covariation signal indicating base-pairing for IS621 are boxed. Complementary nucleotides within covarying regions are highlighted in bold. **c**, Schematic of the in vitro recombination (IVR) reaction with IS621. **d**,**e**, Gel electrophoresis of the IVR LD–RT PCR product (**d**) or LT–RD PCR product (**e**). Results are representative of three technical replicates. Rec, recombinase. **f**, Binding of target and donor DNA sequences by an IS621 RNP containing fluorescently labelled recombinase and ncRNA, using MST. Mean ± s.d. of three technical replicates. **g**, Schematic of the IS621 bridge RNA. The target-binding loop contains the LTG and RTG (blue), and the donor-binding loop contains the LDG and RDG (orange). **h**, Base-pairing model of the IS621 bridge RNA with cognate target and donor DNA.

We next asked whether the ncRNA might assist the recombinase in recognizing the target site or the donor DNA (that is, the IS110 element itself). To assess this, we systematically reconstructed the insertion sites and circular forms of thousands of IS110s (Fig. 2a). An iterative search using a custom structural covariance model of the IS621 ncRNA enabled the prediction of thousands of ncRNA orthologues encoded within LEs^20^ (Methods). We first created a paired alignment of IS110 ncRNAs with their respective target and donor sequences. To assess the possibility of base-pairing between the predicted ncRNAs and their target and donor sequences, we then performed a covariation analysis across 2,201 donor–ncRNA pairs and 5,511 target–ncRNA pairs. We overlaid a base-pairing concordance analysis to identify stretches of the ncRNA that might bind to either the top or the bottom strand of the target or donor DNA^21^ (Supplementary Data 1). Nucleotide sequence covariation would indicate evolutionary pressure to conserve base-pairing interactions between ncRNA positions and target or donor positions.

**Extended Data Fig. 4:**
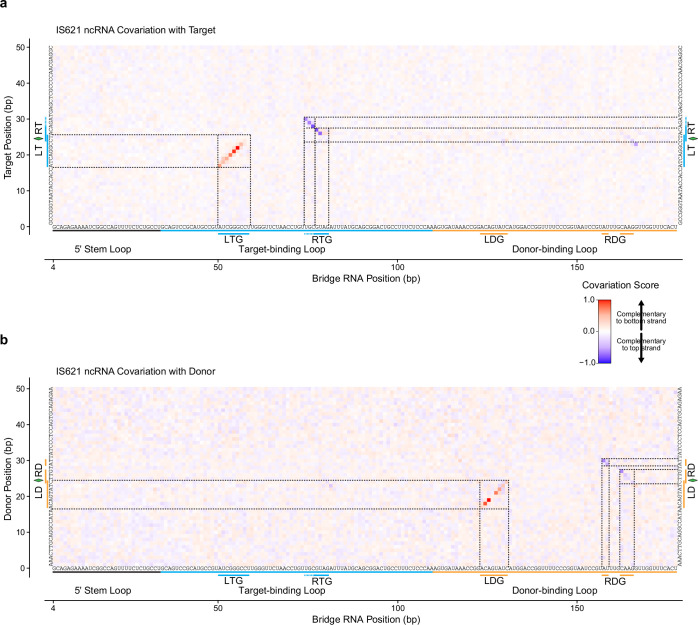
Full ncRNA, target and donor sequence covariation analysis. **a**, Expanded schematic of the target–ncRNA covariation data, zoomed out to include the full target and ncRNA sequences used in the analysis. The corresponding IS621 ncRNA sequence and target used in this study is projected along the x-axis. Covariation scores are coloured according to the sign (+1 or −1) of the column-permuted base-pairing score to better visualize base-pairing patterns. Key features of the bridge RNA are highlighted for clarity. **b**, Expanded schematic of the donor–ncRNA covariation data, zoomed out to include the full donor and ncRNA sequences used in the analysis. Features of the bridge RNA are highlighted for clarity.

This combined analysis clearly indicated potential base-pairing between the two internal loops of the ncRNA and the target and donor DNA sequences, respectively (Fig. 2b and Extended Data Fig. 4a,b). Projecting this covariation pattern onto the canonical IS621 sequence and ncRNA secondary structure, we inferred that the first internal loop might base-pair with the target DNA, whereas the second internal loop might base-pair with the donor DNA. The 5′ side of each loop seems to base-pair with the bottom strand of the target or donor with a stretch of eight or nine nucleotides, whereas the 3′ side of each loop seems to base-pair with the top strand of the target or donor using four to seven nucleotides (Fig. 2b). The strong covariation and base-pairing signal suggest that ncRNA base-pairing with target and donor DNA is a conserved mechanism across diverse IS110 orthologues.

## IS621 ncRNA bridges target and donor DNA

Previous attempts to study IS110 activity have been successful only in IS110 host organisms, with no reports of successful in vitro reconstitution^9,12,15^. We reasoned that the ncRNA could be the missing component required for recombination. To test this, we combined in-vitro-transcribed ncRNA with purified IS621 recombinase and dsDNA oligonucleotides containing target and donor DNA sequences to assess in vitro recombination. Strikingly, we found that the ncRNA is necessary for in vitro recombination, and that the four components (ncRNA, recombinase, target DNA and donor DNA) are sufficient to produce the expected recombination product (Fig. 2c–e and Supplementary Fig. 1). MST also revealed that the recombinase–ncRNA ribonucleoprotein (RNP) complex binds to wild-type (WT) target and donor dsDNA oligos (target *K*_D_ = 13 ± 6 nM; donor *K*_D_ = 77 ± 3 nM), but not to non-complementary DNA molecules (Fig. 2f). Together, these findings indicate that the ncRNA bound by the IS621 recombinase enables sequence-specific binding to both target and donor DNA molecules to facilitate recombination.

We named this ncRNA ‘bridge RNA’, on the basis of its bispecific role in bridging the target and donor DNA molecules for recombination. We refer to the two internal loops of the bridge RNA as the target-binding loop and the donor-binding loop (Fig. 2g). The target-binding loop comprises two key regions that base-pair with the top and bottom strands of the target DNA, respectively: the left target guide (LTG) base-pairs with the left side of the bottom strand of the target DNA (left target; LT), whereas the right target guide (RTG) base-pairs with the right top strand of the target DNA (right target; RT). The donor-binding loop has an analogous architecture, in which a left donor guide (LDG) base-pairs with the bottom strand of the left donor (LD) and a right donor guide (RDG) base-pairs with the top strand of the right donor (RD) (Fig. 2h). Of note, the core dinucleotide is included in each of the base-pairing interactions (LTG–LT, RTG–RT, LDG–LD and RDG–RD), which results in an overlap between the right top and left bottom strand pairings.

**Extended Data Fig. 5:**
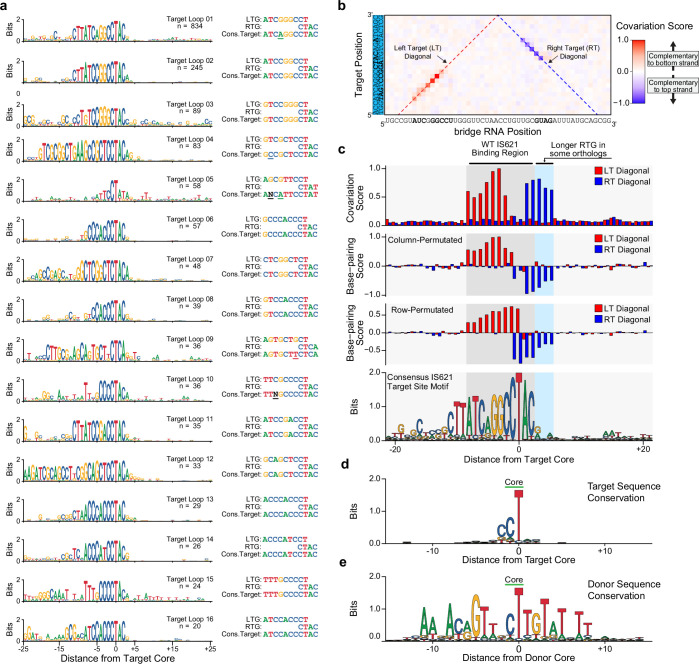
Further details of IS110 covariation and sequence analysis. **a**, Target sequence motifs associated with 16 distinct bridge RNA target-binding loop guide sequences. All unique guides with 20 or more associated target sequences were retained. For each guide, an 11 bp consensus target sequence was constructed by taking the most abundant nucleotide at each position. If two sequences tied as the most abundant nucleotide, they were represented using ambiguous IUPAC code N. Highlighting the mismatching positions between the target guide and the consensus target. **b**, Schematic of the target-bridge RNA covariation analysis presented in Fig. 2b with annotations to indicate the left target (LT) diagonal and the right target (RT) diagonal. The values of different metrics along these diagonals are shown in **c**. **c**, Boundaries of the programmable positions in the target sequence. The top panel indicates the covariation scores along the LT and RT diagonals as generated by CCMpred, which are normalized between 0 and 1. The second panel shows the column-permuted base-pairing score, which is an additional statistic that can be used to identify nucleotide covariation signals while considering both top- and bottom-strand base-pairing. The sign of this score (+1/−1) is multiplied by the covariation score to generate the covariation signals shown in Fig. 2b and Extended Data Fig. 4. The third panel shows the row-permuted base-pairing score. The bottom panel shows a sequence logo for all identified IS621 insertion sites. All these panels are aligned with respect to the core of the target sequence. **d**,**e**, A sequence logo of 5,485 diverse IS621 target (**d**) and donor (**e**) sequences. All unique target and donor sequences that were used as input in the covariation analysis are shown here.

To lend further support to our hypothesis that the bridge RNA target-binding loop guides the selection of the genomic target sequence, we analysed insertion loci across diverse IS110 orthologues. Binning natural IS110s by sequence similarity of their LTG and RTG, we created a consensus genomic target site motif for each LTG, RTG pair. The target motif was highly concordant with the target-binding loop sequences of the bridge RNA (LTG and RTG), with zero to two mismatches in most cases (Fig. 2b, Extended Data Fig. 5a and Supplementary Data 2). Our covariation data further indicated that the RTG of some IS110 orthologues is longer than the RTG for IS621 (Fig. 2b and Extended Data Fig. 5b,c). We also observed evidence of a distinct base-pairing pattern between the RDG and the RD, in which a stretch of nine bridge RNA nucleotides base-pairs discontiguously with a stretch of seven donor DNA bases (Fig. 2b,h).

## Programmable target site selection

**Fig. 3:**
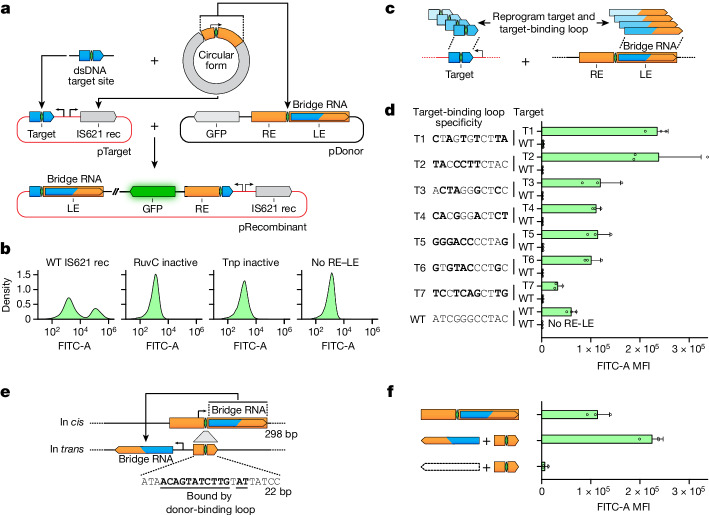
The IS621 target site is reprogrammable and is specified by the bridge RNA. **a**, Schematic representation of the plasmid recombination assay with bridge RNA in *cis*. **b**, GFP fluorescence of *E. coli* after DNA recombination of the plasmid reporter system using catalytic variants of the IS621 recombinase. Plots are representative of three replicates. **c**, Schematic of reprogrammed target and bridge RNA target-binding loop sequences. **d**, GFP mean fluorescence intensity (MFI) of *E. coli* after plasmid recombination using the indicated reprogrammed bridge RNA target-binding loop and target sequences (WT and T1–T7). Bold bases highlight differences relative to the WT target sequence. Mean ± s.d. of three biological replicates. **e**, Schematic of bridge RNA expression in *trans*. **f**, Comparison of recombination efficiency with bridge RNA expressed in *cis* and in *trans*. Mean ± s.d. of three biological replicates.

The base-pairing mechanism of target and donor recognition by the bridge RNA suggests programmability. To assess this, we set up a two-plasmid recombination reporter system in *E. coli*: pTarget encodes the IS621 recombinase, a 50-bp target site and a promoter, and pDonor encodes the RE–LE donor sequence containing the bridge RNA and a promoter-less *gfp*. Recombination of pDonor into pTarget would place *gfp* downstream of the promoter, with successful recombination events detected using flow cytometry (Fig. 3a). Using the WT IS621 donor and target sequences, we detected the expression of GFP and confirmed the expected recombination product using nanopore sequencing (Fig. 3b). Substituting conserved catalytic residues with alanine (Extended Data Fig. 1a,b) abolished recombination, as did substituting pDonor with a version lacking the RE–LE (and therefore lacking the bridge RNA) (Fig. 3b).

**Extended Data Fig. 6:**
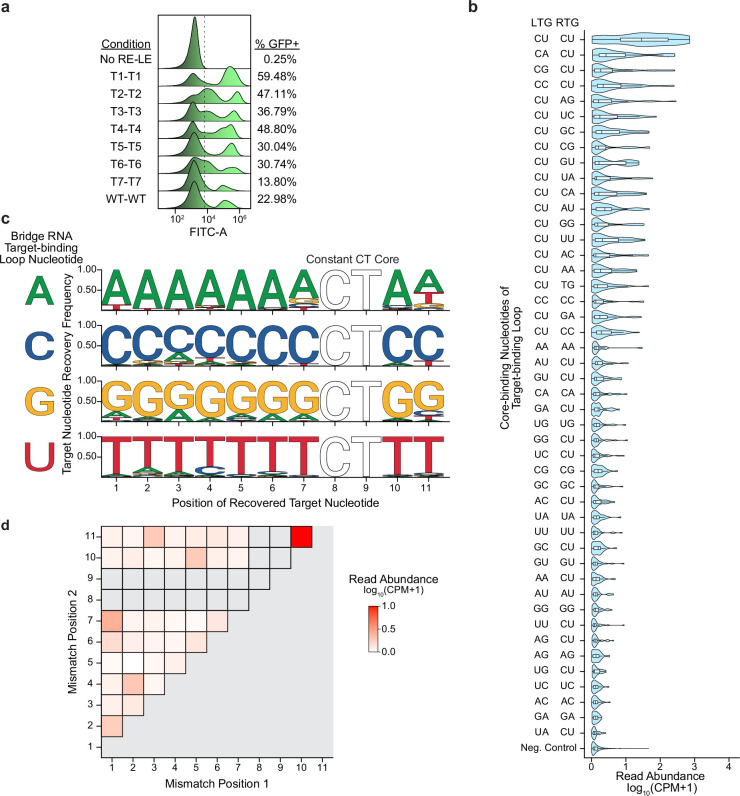
Extended results of RNA target-binding loop and target reprogramming. **a**, DNA recombination in *E*. *coli* with reprogrammed bridge RNAs. The distribution of FITC-A signal for the cell population is shown, with a representative gating strategy for evaluating the percentage of GFP+ cells. T1-T1, T2-T2, etc., represents bridge RNA specificity and provided target, respectively. Plots are representative of 3 replicates featured in Fig. 3d. **b**, Read abundance of oligos with bridge RNA target-binding loop mutations at the positions that bind to the core sequence. The 2 base-pair nucleotides predicted to bind the core in the target-binding loop LTG and RTG were mutated while holding the target and donor CT cores constant and varying the 9 other programmable positions. All tested core mutation combinations shown were tested for 35 different targets, along with a negative control set (n = 1,000) of 9 mismatch target/target-binding loop combinations. **c**, Mismatch tolerance at each position of the 11 bp target sequence. The x-axis shows the target position, with the CT core held constant. The top panel shows the target nucleotide recovery frequency when the target-binding loop contains an A at each guide position, the second panel shows the same but when the target-binding loop contains a C at each position, etc. as a percentage of recovered recombinants at each position. **d**, Double-mismatch tolerance for combinations of positions within the target and target-binding loop. Each cell indicates the average read abundance of oligos that contain double mismatches at the two corresponding positions. The core was held constant. *n* = 800 double-mismatch combinations measured.

We next selected seven target sequences (T1–T7) and designed reprogrammed bridge RNAs with matching target-binding loops (Fig. 3c). These T1–T7 reprogrammed bridge RNAs abrogated recombination with the WT target while enabling high rates of recombination (13.8–59.5% of all cells) with each cognate target sequence (Fig. 3d, Extended Data Fig. 6a and Supplementary Fig. 2). We next asked whether the bridge RNA could be expressed in *trans* rather than within the RE–LE context. We truncated the RE–LE (298 bp) to a 22-bp donor around the core dinucleotide, which eliminated the −35 box of the natural σ^70^ promoter (Fig. 3e). This variant of pDonor did not support recombination into the T5 target plasmid (Fig. 3f) until we supplied the full-length T5 bridge-RNA-encoding sequence in a distinct site on pDonor under the control of a synthetic promoter. The in *trans* bridge RNA increased the total GFP fluorescence signal by nearly twofold compared with the same bridge RNA expressed from the native RE–LE promoter (Fig. 3e,f). Together, these results indicate that the bridge RNA target-binding loop can be reprogrammed to direct target site specificity for DNA recombination in *E. coli*.

**Fig. 4:**
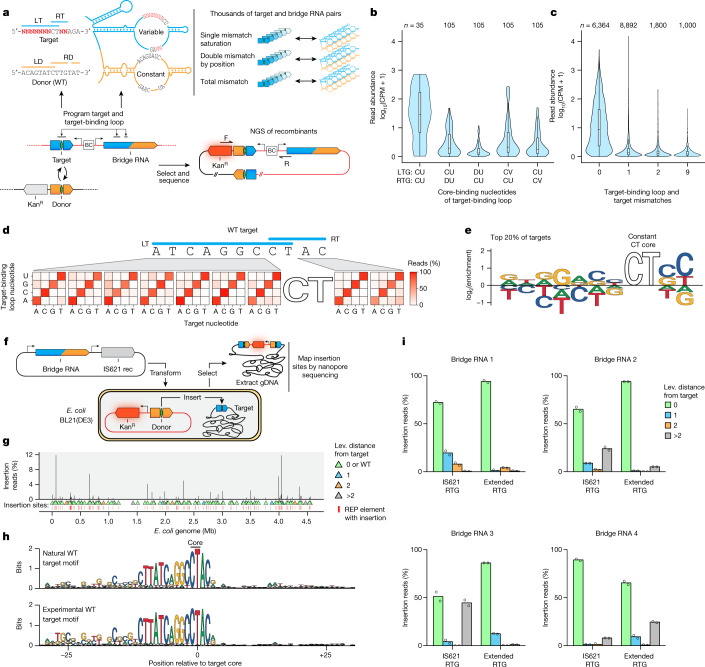
High-throughput characterization of IS621 target specificity shows flexible programmability. **a**, Schematic representation of the target specificity screen. Successful recombination enables the survival of *E. coli* through the expression of a kanamycin resistance cassette (Kan^R^). The target sequence and bridge RNA are separated by a 12-nt barcode (BC). NGS, next-generation sequencing. **b**, Mismatch tolerance of the core dinucleotide. Core-binding nucleotides of the target-binding loop are summarized by IUPAC codes, including D (not C) and V (not U). Average counts per million (CPM) of two biological replicates. Box plots show median (centre line), IQR (box edges) and 1.5 × IQR (whiskers). **c**, Mismatch tolerance between non-core sequences of the target and target-binding loop. Average CPM of two biological replicates. Box plots show median (centre line), IQR (box edges) and 1.5 × IQR (whiskers). **d**, Mismatch tolerance between target and target-binding loop, as indicated by the percentage of total detected recombinants carrying each nucleotide at each position. Average of two biological replicates. **e**, Nucleotide enrichment among the top 20% most efficient matched pairs of targets and target-binding loops. **f**, Schematic of the genome insertion assay in *E. coli*. **g**, Genome-wide mapping of insertions mediated by the WT IS621 bridge RNA. The percentage of total reads mapped to each insertion site is depicted and binned by the number of differences from the intended sites as measured by Levenshtein distance. Average of two biological replicates. **h**, Target site preference of IS621. Sequence logos depict the target site motifs among natural (top, Methods) and experimentally observed (bottom, Fig. 4g) IS621 target sites. **i**, Genomic specificity profile of four reprogrammed bridge RNAs. Two biological replicates.

To comprehensively assess the mismatch tolerance and reprogramming rules of bridge RNAs, we designed an *E. coli* selection screen that links thousands of barcoded pairs of DNA targets and bridge RNAs on a single plasmid. Successful recombination with a WT donor plasmid induces a kanamycin resistance cassette (Kan^R^) for survival (Fig. 4a). Using this approach, we first confirmed that base-pairing between the bridge RNA and both strands of the CT target core sequence was strongly preferred, in line with the high conservation of the CT core sequence in both the target and the donor (Fig. 4b and Extended Data Figs. 5d,e and 6b).

Next, we varied the nine non-core positions of the target and the corresponding positions of the LTG and RTG to assess single and double mismatch tolerance at each position. We observed a strong preference for perfect matches across all nine positions of the target-binding loop and target, and a high degree of reprogramming flexibility at all positions (Fig. 4b–d, Extended Data Fig. 6b,c, Supplementary Table 3 and Supplementary Fig. 3). As expected, double mismatches were even less tolerated than were single mismatches, with bias for certain combinations of mismatch positions (Fig. 4c and Extended Data Fig. 6d). Overall, we show that the target-binding loop is broadly programmable at each position, with a low mismatch tolerance (Fig. 4e).

## Programmable insertion in the *E. coli* genome

**Extended Data Fig. 7:**
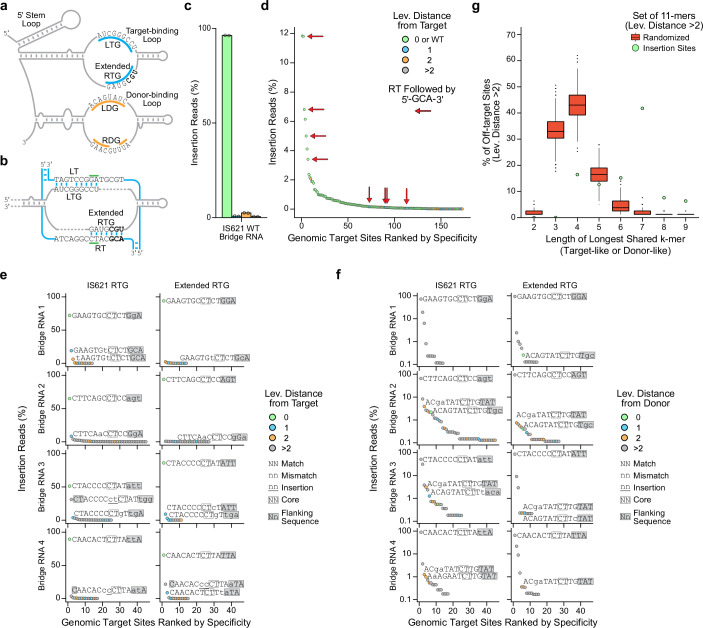
Effect of extended RTGs on the specificity of genome insertion by IS621 bridge RNA and recombinase. **a**, Schematic indicating the bases of the WT bridge RNA which may represent an extended RTG. **b**, Schematic indicating how the target-binding loop of the WT bridge RNA can form more base pairs with a WT 11 bp target sequence flanked on the 5′ end by 5′-GCA-3′. **c**, Genomic specificity profile of the IS621 WT bridge RNAs. Colour indicates the number of differences from the intended sites as measured by Levenshtein distance. Data represent sums of all insertion sites with 0 or WT (ATCAGGCCTAC), 1, 2 or >2 differences from the expected target. **d**, Insertion sites into the *E. coli* genome ranked by abundance. Insertion sites where the RTG flanking bases match the RT flanking bases are indicated with red arrows. **e**, Genomic insertion sites for reprogrammed bridge RNAs displayed in rank order. High frequency insertion sites are highlighted by descriptions of similarity to the intended target sequence. Colour indicates the number of differences from the expected sites as measured by Levenshtein distance. **f**, Genomic insertion sites in **e** recoloured to represent similarity to the WT donor sequence. **g**, Similarity of off-target sites (Lev. distance > 2 from expected target and donor) to the expected target and donor sequences as measured by the length of the longest shared *k*-mer. For comparison, off-target sites were randomly shuffled and the procedure was repeated 1,000 times to generate a null distribution (box plots; showing median (centre line), IQR (box edges), 1.5 × IQR (whiskers) and outliers as points). Observed values are shown as blue points.

To evaluate the genomic site selection and specificity of WT IS621, we measured the insertion of a replication-incompetent plasmid (4.85 kb) bearing the 22-bp WT donor sequence into the *E. coli* genome using the WT IS621 bridge RNA and recombinase (Fig. 4f). After selection, we mapped insertions genome-wide and observed 173 unique insertion sites, with 144 of these insertions occurring within the REP elements that are known^16^ to be targeted by IS621 (Fig. 4g, Supplementary Table 3 and Supplementary Fig. 4). Of all insertion sites, 74.5% (129 sites) matched the naturally observed target sequence (ATCAGGCCTAC), and two more sites exactly matched the specificity encoded by the target-binding loop (ATCGGGCCTAC); together, these accounted for 96.21% of all detected insertions (Extended Data Fig. 7a–c). Our assay therefore recapitulated the specificity of IS621 elements found in nature, including tolerance for a mismatch at position 4 of the target site (Fig. 4h). Structural analysis of the IS621 recombination complex indicates that this mismatch results in a non-canonical rG:dT base pair, which could explain the high frequency of insertions into these target sites^22^.

Further scrutiny of the insertion sites revealed that four of the ten most frequently targeted sites were flanked on the 3′ end of the RT sequence by 5′-GCA-3′—complementary to the 5′-UGC-3′ that occurs immediately 5′ of the RTG in the WT bridge RNA (Fig. 2b and Extended Data Fig. 7a–d). This suggested to us the potential of an extended base-pairing interaction beyond the predicted RTG–RT for IS621 (7 bp instead of 4 bp), which was supported by the observation that some IS110 orthologues naturally encode longer RTGs (Fig. 2b and Extended Data Fig. 5b,c).

To investigate genome-wide insertion specificity, we reprogrammed bridge RNAs to target sequences found only once in the *E. coli* genome. We tested four distinct genomic sites with two bridge RNAs for each: one containing a short 4-bp RTG (IS621 RTG) and one with a long 7-bp RTG (Extended RTG) to directly assess the effect of RTG–RT base-pairing length on specificity. In each case, we found that the expected genomic target site was the most frequently targeted, representing between 51.6% and 94.0% of all detected insertions (Fig. 4i). Off-target insertions were also observed, with individual off-target sites each representing between 0.11% and 31.16% of insertions across all bridge RNAs, with the more frequently detected off-targets typically carrying one or two mismatches with the expected target (Extended Data Fig. 7e).

The extended RTG improved the specificity of insertion into the on-target site from an average of 69.4% (range 51.2–89.4%) to an average of 84.9% (range 65.4–94.0%). It also resulted in markedly fewer insertions into off-target sites for bridge RNA 2 and bridge RNA 3, eliminating 18 out of 45 and 14 out of 25 off-target sites, respectively (Fig. 4i). Notably, some off-target sites seemed to indicate tolerance for insertions in the target sequence, whereas some low-frequency insertions seemed to more closely resemble the 11-bp WT donor sequence, rather than the programmed target (Extended Data Fig. 7e,f). Of the 117 genomic off-target insertion sites detected across the 8 experiments, 102 (87.2%) had the expected CT dinucleotide core, 56 (47.9%) closely resembled the target or donor sequence (Levenshtein distance < 3) and the remaining sites were enriched for long *k*-mer matches to the target or donor sequence (Extended Data Fig. 7g), suggesting that most or all of the detected off-target insertions were bridge-RNA-dependent. In addition to off-target insertions, genomic deletions and inversions between experimentally observed insertion sites were detected in rare cases (allele frequency < 0.05) (Supplementary Note 1). Altogether, these experiments provide evidence of the robust capability of IS621 to specifically insert multi-kilobase cargos into the genome, and offer further insights into the mechanism of recombination.

## Programming the donor specificity of bridge RNAs

**Fig. 5:**
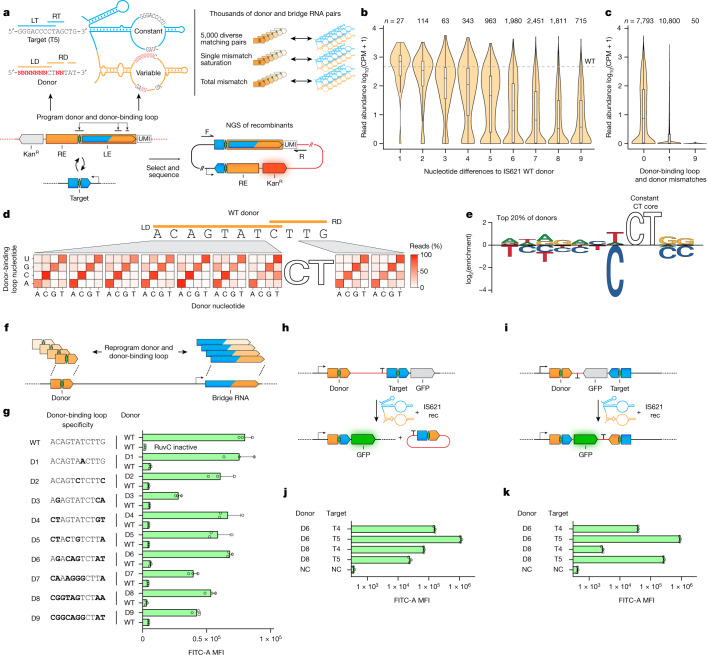
Bridge RNA donor recoding enables fully programmable insertion, inversion and excision. **a**, Schematic representation of the donor specificity screen. A unique molecular identifier (UMI) identifies each paired donor and donor-binding loop. **b**, Reprogrammability of donor sequences by the number of nucleotide differences from the WT donor. WT donor abundance is indicated by the dashed line. Average CPM of two biological replicates. Box plots show median (centre line), IQR (box edges) and 1.5 × IQR (whiskers). **c**, Mismatch tolerance between non-core sequences of the donor-binding loop and donor. Average CPM of two biological replicates. Box plots show median (centre line), IQR (box edges) and 1.5 × IQR (whiskers). **d**, Mismatch tolerance between bridge RNA donor-binding loop and donor by position, as measured by the percentage of total detected recombinants with each indicated mismatch. Average of two biological replicates. **e**, Nucleotide enrichment among the top 20% most efficient matched pairs of donors and donor-binding loops. **f**, Schematic representation of the paired reprogramming of the donor and the donor-binding loop. **g**, Specific recombination using reprogrammed donor and donor-binding loop sequences. Donor sequences are listed on the left, and the bridge RNA is reprogrammed to base-pair with the indicated sequence. Bold bases highlight differences relative to the WT donor sequence. Mean ± s.d. of three biological replicates. **h**, Schematic representation of the programmable excision assay. **i**, Schematic representation of the programmable inversion assay. **j**, Efficient programmable excision of DNA. Pairs of donor and target are denoted. **k**, Efficient programmable inversion of DNA. Pairs of donor and target are denoted. In **j**,**k**, negative control (NC) expresses the reporter and recombinase but no bridge RNA; and data are MFI ± s.d. of three biological replicates.

Among IS621 elements, the donor sequence is more highly conserved than the genomic target sequence, which suggests that the donor-binding loop may be less readily reprogrammed than the target-binding loop (Extended Data Fig. 5d,e). To assess this, we designed a donor specificity screen in which we varied the 7-bp LD and 2 bp of the RD flanking the core dinucleotide, all within the context of a full-length RE–LE expressing the bridge RNA in *cis*. Successful recombination with the T5 target plasmid would induce Kan^R^ expression (Fig. 5a). Analysis of thousands of donor and donor-binding loop pairs revealed that the donor sequence can be fully reprogrammed (Fig. 5b). Similar to the interaction between the target and the target-binding loop of the bridge RNA, LD–LDG mismatches and RD–RDG mismatches were generally poorly tolerated (Fig. 5c, Supplementary Fig. 5 and Supplementary Table 4). Position 7 of the LD was an exception, exhibiting a strong bias against cytosine and therefore appearing to be more mismatch tolerant than other positions (Fig. 5d,e).

**Extended Data Fig. 8:**
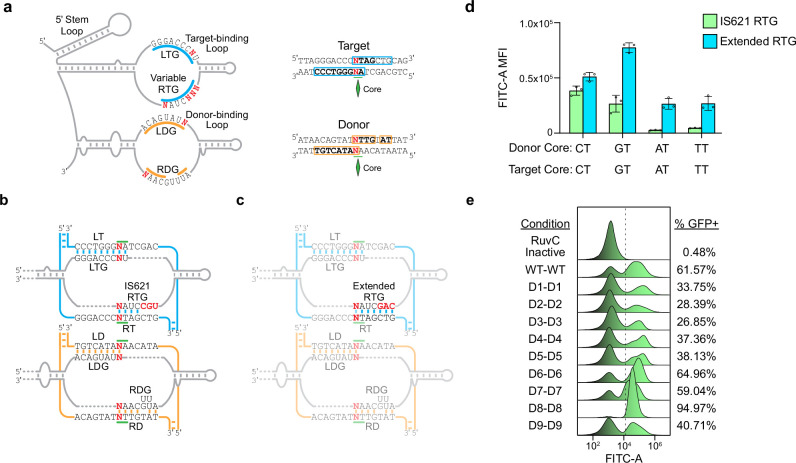
Dual donor and target reprogramming enables core reprogramming and robust DNA recombination. **a**, Schematic representation of core reprogramming with or without RTG extension. The four positions in the guide loops which bind the first base of the core were mutated along with the first base of the core in the target/donor sequences to test if the core sequence can be reprogrammed. The RTG was programmed to allow 7 base binding with the RT or kept as the WT. **b**, Schematic representation of base-pairing between a target-binding loop with the WT IS621 RTG. **c**, Schematic representation of base-pairing between a target-binding loop with a reprogrammed and extended RTG. **d**, Plasmid recombination GFP reporter assay to assess the impact of extended RTG on core programmability. The canonical CT core was mutated to GT, AT, and TT, and tested with the IS621 WT RTG (4 bp) and with an extended RTG (7 bp). MFI ± SD for three biological replicates shown. **e**, DNA recombination in *E. coli* with reprogrammed bridge RNAs. The distribution of FITC-A signal for the cell population is shown, with a representative gating strategy for evaluating the percentage of GFP+ cells. D1-D1, D2-D2, etc., represents bridge RNA specificity and provided donor, respectively. Plots are representative of 3 replicates featured in Fig. 5g.

In these experiments, the core dinucleotide (CT) was held constant, which could limit the sequence space of potential target and donor sites. To address this, we modified the cores of target T5 and the WT donor, along with their associated bridge RNA positions in both loops, from CT to AT, GT or TT (Extended Data Fig. 8a,b). Although non-CT cores were generally less efficient, efficiency was improved by extending the length of RTG–RT base-pairing from 4 bp to 7 bp, informed by our previous results on RTG extension (Fig. 4i and Extended Data Fig. 8c,d).

Next, we investigated the ability of the bridge RNA to combinatorially control the recognition of target and donor sequences simultaneously. Using our in *trans* GFP reporter assay, in which the target-binding loop of the bridge RNA recognizes target T5 (Fig. 3e), we reprogrammed the donor sequence and the donor-binding loop of the bridge RNA to one of nine distinct donor sequences (D1–D9) with varying levels of divergence from the WT donor (Fig. 5f). D1–D9 reprogrammed donor-binding loops supported robust recombination with their cognate donor sequences (26.9–95.0% of all cells) but not with the WT donor (Fig. 5g and Extended Data Fig. 8e). Together, these data show that the bridge RNA allows modular reprogramming of both target and donor DNA recognition.

## Programmable DNA rearrangements

In addition to their use for DNA insertion, recombinases such as Cre have been routinely used for the excision or inversion of DNA sequences. Typically, such approaches require pre-installation of the loxP recognition sites in the appropriate arrangement, with two sites oriented in the same direction resulting in excision, and sites oriented in opposite directions resulting in inversion. Given our understanding of the IS621 insertion mechanism, as well as the reported existence of invertase homologues of IS110s^14,23^, we hypothesized that IS621 recombinases could mediate programmable excision and inversion.

**Extended Data Fig. 9:**
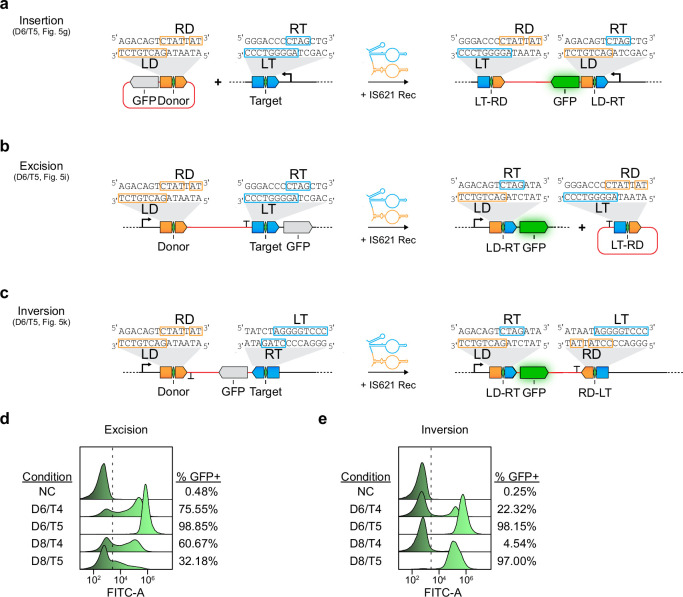
Insertion, excision and inversion using the IS621 bridge recombination system. **a**, Schematic of the insertion reaction. Insertion takes place when the target and donor sequences are on different DNA molecules. The orientation of the insertion can be controlled by the strand placement of the target and the donor. **b**, Schematic of the excision reaction. Excision can occur when the target and donor sequences exist on the same molecule and in the same orientation (i.e. LD and LT are on the same strand). **c**, Schematic of the inversion reaction. Inversion can be catalysed when the target and donor sequences exist on the same molecule, but in the opposing orientation (i.e. on opposite strands). **d**, DNA excision in *E. coli* with reprogrammed bridge RNAs. The distribution of FITC-A signal for the cell population is shown, with a representative gating strategy for evaluating the percentage of GFP+ cells. The donor-target pair is given. Negative control (NC) expresses the reporter with no target or donor, the recombinase, and no bridge RNA. Plots are representative of 3 replicates featured in Fig. 5j. **e**, DNA inversion in *E. coli* with reprogrammed bridge RNAs. The distribution of FITC-A signal for the cell population is shown, with a representative gating strategy for evaluating the percentage of GFP+ cells. The donor-target pair is given. Negative control (NC) expresses the reporter with no target or donor, the recombinase, and no bridge RNA. Plots are representative of 3 replicates featured in Fig. 5k.

We first generated GFP reporter systems for both excision and inversion (Fig. 5h,i and Extended Data Fig. 9a–c). Testing the same four pairs of donor and target recognition sites in both reporters, we showed that both excision and inversion occur robustly and in a programmable manner (32.2–98.9% and 4.54–98.2% of all cells, respectively) (Fig. 5j,k and Extended Data Fig. 9d,e). Overall, the ability of IS110 recombinases and their bridge RNAs to insert, excise and invert DNA in a programmable and site-specific manner enables remarkable control over multiple types of DNA rearrangements with a single unified system.

## Diverse IS110s encode bridge RNAs

**Fig. 6:**
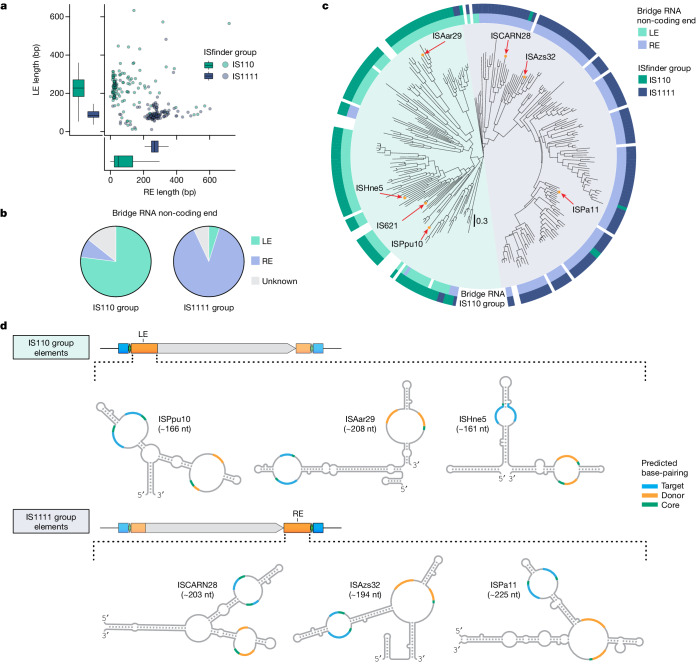
IS110 subfamilies encode distinct and diverse bridge RNA secondary structures in different non-coding end sequences. **a**, Non-coding end length distribution for IS110 and IS1111 group elements. Box plots show median (centre line), IQR (box edges) and 1.5 × IQR (whiskers). **b**, Location of predicted bridge RNA for IS110 and IS1111 group elements. **c**, Phylogenetic tree of the 274 IS110 recombinases catalogued by ISfinder. **d**, Bridge RNA consensus structures from six diverse IS110 elements. Secondary structures are shown with internal loops coloured according to the sequence that they complement: target (blue), donor (orange) or core (green).

**Extended Data Fig. 10:**
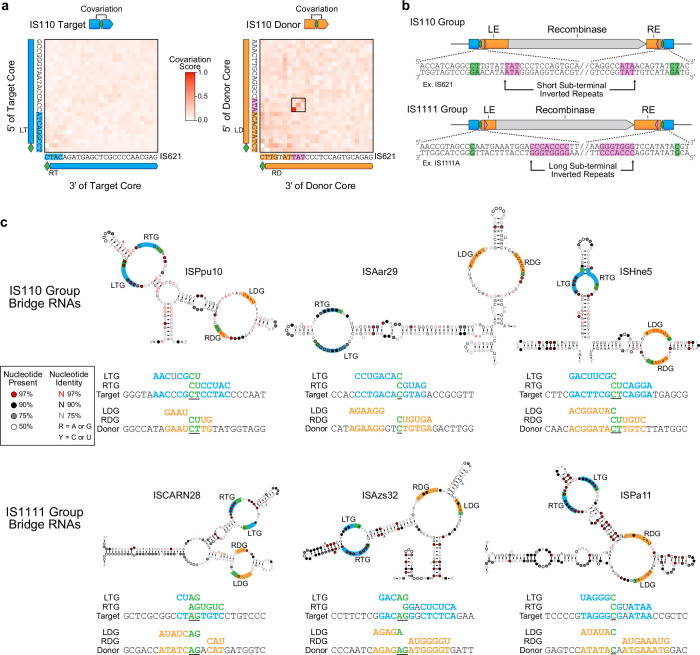
Detailed analysis of diverse bridge RNA sequences and their predicted target and donor binding patterns. **a**, Covariation analysis of IS110 donor sequences identifies a short STIR. Target and donor sequences were analysed using the same covariation analysis introduced in Fig. 2b. Target sequences have no notable covariation signal while donor sequences have a prominent 3-base covariation signal that corresponds with an LT-flanking ATA tri-nucleotide and a RD-flanking TAT tri-nucleotide. **b**, Schematic depicting sequence features of IS110 and IS1111 group elements. IS110 are characterized by long LEs, short REs, and short STIRs. IS1111 are characterized by short LEs, long REs, and long STIRs. **c**, Six diverse bridge RNAs and their predicted binding patterns. The bridge RNA consensus structures shown are the same as those presented in Fig. 6d, but with more detail. Secondary structures are shown with internal loops coloured according to the sequence that they complement - target (blue), donor (orange), or core (green). Three members of each IS110 group are shown. For each of the six sequence elements catalogued in ISfinder - ISPpu10, ISAar29, ISHne5, ISCARN28, ISAzs32, and ISPa11 - IS element boundaries were inspected to identify possible base-pairing between the loops, the targets, and the donors. Under each structure, the predicted LTG, RTG, target, LDG, RDG and donor are all shown and aligned with respect to the core (underlined in black).

Finally, we investigated whether the bridge RNA is a general feature of the IS110 family. The IS110 family is divided into two groups: IS110 (which includes IS621) and IS1111. IS1111 elements also encode DEDD recombinases, but have been categorized into a separate group on the basis of the presence of sub-terminal inverted repeat sequences (STIRs) that range in length from 7 to 17 bp^8,10,13^. We examined our covariation analysis of IS110 group termini and identified a short 2–3-bp STIR pattern that flanks the programmable donor sequence, suggesting an evolutionary relationship with the longer STIRs of IS1111 elements (Extended Data Fig. 10a,b). Amongst all IS110 and IS1111 elements annotated in the ISfinder database, we found that IS1111 elements have much longer REs than LEs—in contrast to the IS110 subgroup, in which the LE is significantly longer than the RE (Fig. 6a).

Using RNA structural covariance models, we predicted a bridge RNA in 85.7% of IS110s and 93.0% of IS1111s (Fig. 6b). The vast majority of IS110 group members appeared to encode a bridge RNA within the LE, whereas IS1111 group members appeared to encode a bridge RNA within the RE. This is consistent with a previous report that correlated target site preference with sequence conservation in the RE of IS1111 elements and, on this basis, speculated that an RNA might be involved in target site selection^24^. Notably, the location of the bridge RNA closely predicted the phylogenetic relationship between IS110 and IS1111, which strongly suggests that these two groups emerged from a common ancestor in which the bridge RNA translocated between the ends of the element and the length of the STIR was modified (Fig. 6c and Supplementary Table 5).

We predicted bridge RNA structures and manually inspected the loops of six diverse IS110 and IS1111 elements for evidence of complementarity with their cognate target and donor sequences. This analysis yielded diverse structures with clear evidence of a base-pairing pattern (8–14 nt) between internal bridge RNA loops and DNA targets and donors (Fig. 6d and Extended Data Fig. 10c). Of note, in many IS1111 orthologues, the predicted bridge RNA has potential donor-binding nucleotides in a multi-loop structure rather than the simple internal loop observed for IS621 and other members of the IS110 group. Altogether, we conclude that the IS110 family encodes diverse predicted bridge RNAs that direct sequence-specific and programmable recombination between target and donor sequences.

## Discussion

Non-coding RNA molecules that specify a nucleic acid target are central to both prokaryotic and eukaryotic life. Nucleic acid guides are a widely used mechanism in fundamental biological processes; for example, the tRNA anticodons that govern ribosomal translation; small interfering RNAs and microRNAs of RNA interference; CRISPR RNAs of CRISPR–Cas immunity; and small nucleolar RNAs (snoRNAs) for gene regulation. The bridge RNA that we discovered in this work is the first example, to our knowledge, of a bispecific guide molecule that encodes modular regions of specificity for both the target and the donor DNA, coordinating these two DNA sequences in close proximity to catalyse efficient recombination. Bridge RNAs encode all of this complex molecular logic in a remarkably compact (around 150–250-nt) sequence along with their single effector recombinase (around 300–460-aa) partner.

IS110 targeting is achieved using internal binding loops that are reminiscent of tRNA hairpin loops or snoRNA internal loops, distinct from the terminal binding sequences of CRISPR–Cas or Argonaute guide RNAs. Each RNA loop encodes segments that base-pair with staggered regions of the top and bottom strand of each cognate DNA binding partner, in contrast to the single-strand base-pairing mechanisms of known RNA-guided systems. Furthermore, the RNA-guided self-recognition of the IS110 element in donor form illustrates a previously unobserved mechanism of DNA mobility.

Mobile genetic elements have been shaped throughout evolution to insert, excise, invert, duplicate and otherwise rearrange DNA molecules. Bridge RNAs enable IS110 recombinases to exploit the inherent logic of RNA–DNA base-pairing, directly bypassing the complex target site recognition codes of other known transposases and recombinases, which depend on extensive protein–DNA or short single-stranded DNA–DNA interactions that offer much less opportunity for straightforward programmability^25–28^. The IS110 family is evolutionarily diverse and widespread across prokaryotes, providing a rich landscape for further functional insights. In our initial survey of diverse IS110 orthologues, we uncovered a variety of bridge RNA structures and lengths, suggesting that there is considerable mechanistic diversity both between and within each of the two major IS110 and IS1111 subfamilies.

Our accompanying cryo-electron microscopy analysis of the IS621 recombinase in complex with bridge RNA, target DNA and donor DNA, captured in several stages of the recombination reaction, is copublished with this paper^22^. Together, our two studies detail the unique mode of dual-strand recognition of the target and donor DNA through programmable base-pairing interactions with the bridge RNA. The synaptic complex structures illustrate how two recombinase dimers associate with the target-binding loop and the donor-binding loop of bridge RNAs, coming together to form an adaptable recombination complex (ARC) with composite subunit-spanning active sites when both target and donor DNA are engaged by the ARC system. This elegant licensing mechanism enables nicking and exchange of the top strands between the donor and target, resulting in a Holliday junction intermediate that is resolved by the cleavage of the bottom strands. Together, our genetic, mechanistic, computational and structural characterization of the bridge recombination system lays the foundation for protein and RNA engineering efforts to improve and optimize its capabilities.

Guide RNAs are underpinning a technological revolution in programmable biology^29–35^. The direct enzymatic activity of stand-alone, naturally occurring programmable RNA-guided proteins has been notably limited to the endonuclease function^30,36^. Successive generations of programmable nucleases and nickases have advanced the prevailing genome-editing method from the original homology-based capture of a DNA donor^37^ to the targeted stimulation of donor insertion, all of which require a complex interplay with endogenous DNA repair processes^31,34,38–40^. Functional diversification of these systems beyond nucleic acid binding or cleavage has generally required the recruitment or fusion of additional effector proteins, resulting in increasingly large and intricate engineered genome-editing fusions^41,42^. The IS110 bridge system, in contrast, uses a single and compact RNA-guided recombinase that is necessary and sufficient for direct DNA recombination (Fig. 2d,e). Modular reprogramming of target and donor recognition by the bispecific bridge RNA uniquely enables the three fundamental DNA rearrangements of insertion, excision and inversion for manipulating large-scale DNA sequences and overall genome organization. With further exploration and development, we expect that the bridge recombination mechanism will spur a third generation of programmable RNA-guided tools beyond RNA interference- and CRISPR-based mechanisms to enable a new frontier of genome design.

## Methods

### Development of metagenomic and genomic sequence database

A custom sequence database of bacterial isolate and metagenomic sequences was constructed by aggregating publicly available sequence databases, including NCBI, UHGG^43^, JGI IMG^44^, the Gut Phage Database^45^, the Human Gastrointestinal Bacteria Genome Collection^46^, MGnify^47^, Youngblut et al. animal gut metagenomes^48^, MG-RAST^49^ and Tara Oceans samples^50^. The final sequence database included 37,067 metagenomes, 274,880 bacterial and archaeal metagenome-assembled genomes, 855,228 bacterial and archaeal isolate genomes and 185,140 predicted viral genomes.

### Analysis of conserved residues in IS110 protein sequences

Genomic sequences were annotated using Prodigal^51^ to identify coding sequences. All unique protein sequences were then combined into a single FASTA file and clustered at 30% sequence identity using mmseqs2 (ref. ^52^). Two Pfam domains DEDD_Tnp_IS110 (PF01548) and Transposase_20 (PF02371) were used to search against these clustered representative proteins using the hmmsearch tool in the hmmer package^53^. DEDD_Tnp_IS110 was used to identify the RuvC-like domain, and Transposase_20 was used to identify the Tnp domain. All members of the matched 30% identity clusters were then extracted, and the same IS110 Pfam domain significance thresholds were applied to filter these candidates. Next, only proteins that met *E* < 1 × 10^−3^ for both domains were retained. Next, RuvC-like domains were only retained if they were between 125 and 175 aa in length, and Tnp domains were only retained if they were between 60 and 110 aa in length. Any sequences with ambiguous residues were removed. Protein domains were then clustered at 90% using mmseqs (‘easy-cluster --cluster-reassign -c 0.8 --min-seq-id 0.9 --cov-mode 0’). Cluster representatives were then aligned using hmmalign (‘--trim --amino’)^53^. Alignment columns with more than 50% gaps were removed, and the alignments were visualized using ggseqlogo in R^54^.

### Phylogenetic analysis of IS110 transposases

A phylogenetic analysis of IS110 transposases was also performed. Full-length IS110 proteins were clustered at 90% identity using the mmseqs2 easy-cluster algorithm (‘--cluster-reassign -c 0.85 --min-seq-id 0.9 --cov-mode 0’)^52^. Next, using the identified 90% protein sequence clusters, a representative from each cluster was selected that was closest to the 80th percentile in total length. This resulted in a curated set of 90% identity cluster representatives. Next, 90% identity cluster representatives were clustered at 30% identity across 70% of the aligned sequences using the mmseqs2 easy-cluster algorithm (‘--cluster-reassign -c 0.70 --min-seq-id 0.30 --threads 96 --cov-mode 0’). This resulted in 1,686 30% identity cluster representatives. RuvC-like and Tnp-like domains were extracted from these proteins using the corresponding Pfam pHMM models and hmmsearch^53^. These extracted domains were then individually aligned using hmmalign (‘--amino --trim’) and concatenated into a paired alignment. All pairwise percentage identity values were calculated for this alignment, and redundant sequences were removed using a 60% identity cut-off, resulting in 1,054 aligned sequences. A phylogenetic tree was then constructed using iqtree2 v.2.1.4-beta, with all default parameters^55^, midpoint rooted and visualized in R with ggtree^56^. Additional metadata about each sequence was mapped onto the tree, including host kingdom and phylum, ISfinder group and notable orthologues.

Curated ISfinder transposases were analysed separately to produce another phylogenetic tree. IS110 transposase sequences were extracted from the database available through the prokka software package^57^. Only IS110 transposases of more than 250 aa were retained. Protein sequences were then clustered using mmseqs2 (‘easy-cluster -c 0.5 --min-seq-id 0.9 --threads 8 --cov-mode 0’)^52^. Cluster representatives were then aligned using mafft-ginsi (‘--maxiterate 1000’)^58^. Alignment columns with more than 50% gaps were removed. A phylogenetic tree was then constructed using iqtree2 v.2.1.4-beta with all default parameters^55^.

### Analysis of LE and RE lengths across IS110 elements

Sequence coordinate information about individual IS elements was collected through the ISfinder web portal^59^. This included information about the total length of each IS element, as well as the start and end coordinates of the recombinase CDS. The LE non-coding length was calculated from the CDS coordinates for each IS110 element as the distance between the 5′ terminus and the start of the CDS, and the RE non-coding length was calculated as the distance between the end of the CDS and the 3′ terminus. Tn3 family elements were excluded owing to highly variable passenger gene content.

### Predicting IS110 element boundaries

To identify the boundaries of each element, an initial search was conducted using comparative genomics to identify putative pre-insertion and post-insertion examples within the metagenomic sequence database. IS110 protein candidates were clustered at 30% identity using mmseqs2 (ref. ^52^), and within each cluster all relevant genomic loci were identified. Nucleotide sequences were then extracted from the database by adding 1,000 base pairs to the 5′ and 3′ ends of the IS110 CDS, and extracting the complete intervening sequence. These IS110 loci were then separated into ‘batches’ on the basis of 90% identity protein clusters. These batches were then searched against up to 40 metagenomic or isolate samples in the custom database, prioritizing samples that already contained related recombinases. Putative pre-insertion sites were identified if the distal ends of the loci aligned by BLAST to a contiguous sequence^60^, but the IS110 CDS did not. Precise boundaries of the IS110 element were then predicted using a modified method similar to what was implemented by the previously published tool MGEfinder^61^. Core sequences were identified as repeated sequences near the end of the predicted element. Next, an iterative BLAST search was used to extend IS110 element boundary predictions beyond those that could be detected by identifying pre-insertion sites. IS110 elements were searched using BLAST against all IS110 loci. Hits were retained only if both ends of the element aligned, and if the core was concordant between query and target. This then generated a new set of IS110 elements and their boundaries, which were recycled as query sequences, and the search was repeated for another iteration. This repeated for 36 iterations before convergence (no new IS110 elements were found). The combined set of IS110 boundaries were kept for further analysis.

### Identification of bridge RNA consensus structures

A pipeline was developed to identify conserved RNA structures in the sequences immediately flanking the recombinase CDS. First, the IS621 protein sequence was searched against the complete IS110 database for orthologues using blastp (‘-max_target_seqs 1000000 -evalue 1e-6’). Only hits that were at least 30% identical at the amino acid level with 80% of both sequences covered by the alignment were retained. Up to 2,000 unique proteins were then selected in order of descending percentage amino acid identity. Flanking sequences for the corresponding proteins were then retrieved from the database, with flanking sequences defined as a 5′ flank of up to 255 bp (including 50 bp of 5′ CDS) and a 3′ flank of up to 170 bp (including 50 bp of the 3′ CDS). These flanks were then further filtered to exclude sequences that were more than 35 bases shorter than the target flank lengths. Sequences were filtered to exclude those with ambiguous nucleotides. Protein sequences were then clustered using mmseqs2 easy-linclust with a minimum percentage nucleotide identity cut-off of 95% across 80% of the aligned sequences, and one set of flanks for each representative was retained. Flanking sequences were then clustered at 90% nucleotide identity across 80% of the aligned sequences, and only one representative flanking sequence pair per cluster was retained. Then, up to 200 sequences were selected in order of decreasing percentage identity shared between the IS621 protein sequence and their corresponding orthologue protein sequence. The remaining sequences were then individually analysed for secondary RNA structures using linearfold^62^. Sequences were then aligned to each other using the mafft-xinsi (IS621 orthologue sequences) or mafft-qinsi (all other ISfinder elements) alignment algorithms and parameter --maxiterate 1000 (ref. ^58^). Alignment columns with more than 50% gaps were removed. The conserved RNA secondary structure was then projected onto the alignment, and manually inspected to nominate bridge RNA boundaries. This region was exported as a separate sequence alignment file, and a consensus RNA secondary structure was predicted using ConsAlifold^63^. This structure was then visualized using R2R^64^. This same pipeline was used to analyse hundreds of other IS110 elements, resulting in diverse predicted secondary structures. For visualization purposes, consensus secondary structures with minimally structured terminal ends were trimmed to the primary structured sequence. These consensus structures were converted into covariance models using Infernal^20^, and these were then searched across thousands of sequences to identify putative bridge RNAs^20^.

### Nucleotide covariation analysis to identify bridge RNA guide sequences

To identify programmable guide sequences in the bridge RNA of the IS621 element, the following approach was taken. First, the IS621 protein sequence was searched against our collection of IS110 recombinase proteins with predicted element boundaries using blastp. Next, only alignments that met a cut-off of 20% amino acid identity across 90% of both sequences were retained. Next, a covariance model of the bridge RNA secondary and primary sequence was used to identify homologues of the bridge RNA sequence in the non-coding ends of these orthologous sequences^20^. Fifty nucleotide target and donor sequences were extracted centred around the core. For elements with multiple predicted boundaries, boundaries with a CT dinucleotide core were prioritized. Next, elements that were identified at earlier iterations in our boundary search were prioritized. Next, elements that were similar in length to the known IS621 sequence element were prioritized. Only one element per unique locus was retained. Alignments were further filtered to remove redundant examples by clustering targets or donors and bridge RNA sequences at 95% identity, taking one representative per pair and then taking at most 20 examples for each 95% identity bridge RNA cluster. Predicted bridge RNA sequences were then aligned using the cmalign tool in the Infernal package^20^. Two paired alignments were then generated that contained concatenated target and bridge RNA sequences, and concatenated donor and bridge RNA sequences. These alignments were then further filtered to remove all columns that contained gaps in the IS621 bridge RNA sequence. These alignments were then analysed using CCMpred (‘-n 100’) to identify covarying nucleotides between targets or donors and bridge RNA sequences^65^. These covariation scores were normalized by min-max normalization and multiplied by the sign of the column-permuted base-pairing concordance score (see next paragraph), with +1 corresponding to bottom-strand base-pairing and −1 corresponding to top-strand base-pairing. The signal was visualized as a heat map and interactions were identified within the two internal loops of the bridge RNA, leading to the proposed model for bridge RNA target or donor recognition. The same covariation analysis was performed on the donor alone, leading to the identification of short STIR sequences for IS110 elements.

A separate analysis was performed on the same paired alignment used in the covariation analysis to determine whether certain pairs of nucleotides were biased toward base-pairing. The observed concordance was first calculated for each pair of columns as:

$${C}_{ij}\,=\frac{{\rm{a}}{\rm{b}}{\rm{s}}{\rm{m}}{\rm{a}}{\rm{x}}({\sum }_{k=1}^{n}{\rm{C}}{\rm{h}}{\rm{e}}{\rm{c}}{\rm{k}}{\rm{E}}{\rm{q}}{\rm{u}}{\rm{a}}{\rm{l}}({s}_{ki},\,{t}_{kj}),{\sum }_{k=1}^{n}{\rm{C}}{\rm{h}}{\rm{e}}{\rm{c}}{\rm{k}}{\rm{C}}{\rm{o}}{\rm{m}}{\rm{p}}{\rm{l}}{\rm{e}}{\rm{m}}{\rm{e}}{\rm{n}}{\rm{t}}{\rm{a}}{\rm{r}}{\rm{y}}({s}_{ki},\,{t}_{kj}))}{n},$$

where *C* is the concordance score, *i* refers to the first column (or position), *j* refers to the second column, *n* refers to the total number of rows (sequences) in the alignment, *s*_*ki*_ refers to the nucleotide in bridge RNA sequence *k* at position *i* and *t*_*kj*_ refers to the nucleotide in target (or donor) sequence *k* at position *j*. absmax(*a,b*) is a function that returns the value with the largest absolute magnitude, CheckEqual(*a,b*) is a function that returns one when *a* = *b* and 0 otherwise and CheckComplementary(*a,b*) is a function that returns −1 if *a* and *b* are complementary nucleotides and 0 otherwise. All positions in which the nucleotide is a gap in either sequence are ignored and discounted from *n*. All observed values of *C*_*ij*_ are then compared with two different null distributions of *C*_*ij*_ scores. The first is generated by randomly permuting the rows of the bridge RNA alignment 1,000 times and recalculating *C* for each permutation, and the second is generated by randomly permuting the columns of the bridge RNA alignment 1,000 times and recalculating *C*. The mean and standard deviation of these permuted *C* distributions are then used to convert the observed *C* scores into *z*-scores, and positive and negative values are then separately min-max normalized to maintain the −1 to 1 scale. The sign of this score is then used to project base-pairing information onto the covariation scores as generated by CCMpred.

### Small RNA-seq of IS110 bridge RNAs

BL21(DE3) *E. coli* were transformed with plasmids bearing a concatenated RE–LE sequence and plated on an LB agar plate with appropriate antibiotics. A single colony was picked and grown in terrific broth (TB) to an optical density (OD) of 0.5. RNA isolation was performed using the Direct-zol RNA Miniprep kit (Zymo Research). RNA was prepared for small RNA-seq according to the following protocol. In brief, no more than 5 µg total RNA was treated with DNase I (NEB) for 30 min at 37 °C then purified using the RNA Clean & Concentrator-5 kit. Ribosomal RNA was depleted from samples using the Ribo-Zero Plus rRNA Depletion kit (Illumina) and purified using the RNA Clean & Concentrator-5 kit. Depleted RNA was treated with T4 PNK for six hours at 37 °C, supplementing with T4 PNK and ATP after six hours for one additional hour. RNA was purified using the RNA Clean & Concentrator-5 kit and subsequently treated with RNA 5′ polyphosphatase (Lucigen) for 30 min at 37 °C. RNA was purified with the RNA Clean & Concentrator-5 kit, and the concentration was measured by NanoDrop. NGS libraries were prepared using the NEBNext Multiplex Small RNA Library Prep Kit (NEB) according to the manufacturer’s protocol. Libraries were sequenced on an Illumina MiSeq using a 2×150 Reagent kit (v.2).

### Analysis of small RNA-seq data

Demultiplexed fastq files were cleaned and merged using BBtools (bbduk and bbmerge), respectively^66^. Merged fastq files were aligned to the RE–LE-bearing plasmid using bwa-mem^67^. Small RNA-seq coverage was normalized according to the maximum read depth observed for each orthologue across the entire RE–LE plasmid.

### In vitro transcription of bridge RNAs

In vitro transcription was performed on a linear DNA template using the HighScribe T7 High Yield RNA Synthesis Kit (New England Biolabs) as per the manufacturer’s instructions. The DNA template was prepared by cloning into a pUC19 backbone and the plasmid was linearized using the SapI restriction enzyme (NEB) and purified using DNA Clean & Concentrate (Zymogen). After in vitro transcription, RNA was purified using the Monarch RNA Cleanup kit. Where necessary, bridge RNA was further purified by denaturing polyacrylamide gel electrophoresis, extracted from the gel using UV shadowing and recovered by ethanol precipitation.

### IS621 protein preparation

The IS621 recombinase gene was human codon optimized and cloned into a modified pFastBac expression vector (Addgene, 30115), which includes an N-terminal His_6_-tag, a TwinStrep-tag and a tobacco etch virus (TEV) protease cleavage site. To express IS621 recombinase protein Sf9 cells (ATCC, CRL-1711) were cultured in Sf-900 III SFM medium (Thermo Fisher Scientific) supplemented with 10 µg μl^−1^ gentamicin and 5% heat-inactivated fetal bovine serum (Gibco). For baculovirus production, recombinant bacmids were first generated by transforming MAX Efficiency DH10Bac competent cells (Thermo Fisher Scientific) with the pFastBac construct. Site-specific recombination between pFastBac and the baculovirus shuttle vector was then confirmed by PCR and Sanger sequencing. For large-scale protein expression, a high-titre P1 recombinant (pFastBac) baculovirus stock was used; cells were infected with pFastBac baculovirus at a multiplicity of infection of 5 plaque-forming units per cell at a cell density of 3 × 10^6^ cells per ml and grown in suspension culture at 28 °C. Cells were collected by centrifugation (300*g*, 15 min, 4 °C) 48 h after infection and lysed by sonication in buffer containing 20 mM Tris-HCl, pH 7.5, 1 M NaCl, 2 mM MgCl_2_, 1 mM dithiothreitol (DTT), 10% glycerol and 2% Triton-X, supplemented with cOmplete EDTA-free mini protease inhibitor cocktail (Roche). Then the lysate was clarified by ultracentrifugation at 45,000*g* and filtered through a 0.45-µm PVDF syringe filter (Millipore Sigma). The supernatant was applied to a 5-ml Strep-Tactin Superflow high-capacity FPLC column (IBA Lifesciences) and washed with 20 column volumes of wash buffer containing 20 mM Tris-HCl, pH 7.5, 0.5 M NaCl, 2 mM MgCl_2_, 1 mM DTT and 10% glycerol, and the protein was eluted with wash buffer containing 80 mM biotin. Eluted protein was concentrated using a 10-kDa molecular weight cut-off (MWCO) ultracentrifugal concentrator (Millipore Sigma) at 4 °C and the His-TwinStrep-tag was cleaved using TEV protease (NEB) at 37 °C for 4 h. His-TwinStrep-tag cleaved protein was then applied to a 5 ml HisTrapFF Crude immobilized metal affinity column (Cytiva) equilibrated with wash buffer containing 20 mM Tris-HCl, pH 7.5, 0.5 M NaCl, 2 mM MgCl_2_, 1 mM dithiothreitol (DTT) and 10% glycerol. Wash fractions expected to contain His-TwinStrep-tag-removed IS621 recombinase protein were collected and bound protein was eluted using wash buffer containing 0.5 M imidazole. Notably, IS621 recombinase remained bound to the HisTrapFF column despite His-TwinStrep-tag removal and eluted in the presence of high imidazole. Finally, elution fractions containing recombinant protein were concentrated using a 10-kDa-MWCO ultracentrifugal concentrator (Millipore Sigma) and buffer exchanged during centrifugation into size-exclusion chromatography (SEC) buffer containing 20 mM Tris-HCl, pH 7.5, 0.5 M NaCl, 2 mM MgCl_2_, 1 mM DTT and 10% glycerol. SEC was performed using a Superdex 200 Increase 10/300 GL column (Cytiva) to further purify the protein, and the peak fractions were collected, concentrated as described above and stored at −80 °C until use.

### Microscale thermophoresis (MST)

MST was performed using a Monolith NT.115^Pico^ series instrument (NanoTemper Technologies). IS621 recombinase was labelled for MST using the RED-MALEIMIDE 2nd Generation cysteine reactive kit (NanoTemper Technologies) as per the manufacturer’s instructions. Labelled protein was eluted in a buffer containing 20 mM Tris-HCl, 500 mM NaCl, 5 mM MgCl_2_, 1 mM DTT and 0.01% Tween20, pH 7.5. To determine the affinity of recombinase for RNA, 20 nM recombinase was incubated with a dilution series (2,500–0.076 nM) of purified LE-encoded ncRNA or a scrambled RNA of equivalent length. MST was performed at 37 °C using premium capillaries (NanoTemper Technologies) at 30% LED excitation and medium MST power. Data were analysed using the NanoTemper MO.affinity analysis (v.3.0.5) software package and raw data were plotted on GraphPad Prism (v.10.2.0) for visualization. The binding affinities of the IS621 RNP for donor and target DNA, as well as for donor and target DNA containing scrambled LD–RD and LT–RT regions, were determined using the MST tertiary binding function. Single-stranded DNA was purchased from IDT and annealed in buffer containing 10 mM Tris pH 8.0, 5 mM MgCl_2_ and 5 mM KCl. For MST, 20 nM RNP consisting of labelled IS621 recombinase and LE-encoded ncRNA were incubated with a dilution series of duplexed donor or target DNA oligonucleotides (10 µM to 0.076 nM). MST was performed at 37 °C using premium capillaries (NanoTemper Technologies) at medium MST power with the LED excitation power set to automatic (excitation ranged from 20% to 50%).

### In vitro recombination assay

The in vitro activity of IS621 recombinase was evaluated by incubating 10 µM IS621 with 20 µM LE-encoded ncRNA and 0.5 µM duplexed target and donor DNA oligonucleotides (Supplementary Information) in buffer containing 20 mM Tris-HCl, 300 mM NaCl, 5 mM MgCl_2_, 1 mM DTT, 0.05 U µl^−1^ SUPERase•In RNase Inhibitor (Invitrogen) at 37 °C for two hours. Reactions were then treated with 40 µg Monarch RNaseA (NEB) for one hour and then treated with 1.6 units of Proteinase K (NEB) for a further hour before clean-up of DNA with AMPure XP Beads (Beckman Coulter) using a 2× bead ratio. To detect recombination products, 0.5 µl of the purified reaction product was PCR-amplified with primers designed to amplify the LT–RD and LD–RT recombination products. PCR products were visualized by running PCR reactions on 8% TBE gel (Invitrogen) and staining with SYBR Safe (Thermo Fisher Scientific), and were imaged on a ChemiDoc XRS+ (Bio-Rad). PCR products were sequenced using Oxford Nanopore sequencing (Primordium Labs).

### Plasmid recombination assay in *E. coli*

BL21(DE3) cells (NEB) were co-transformed with a pTarget plasmid encoding a target sequence and a T7-inducible IS621 recombinase and a pDonor plasmid encoding a bridge RNA, a donor sequence and a GFP CDS upstream such that after recombination into pRecombinant GFP, expression would be activated by the synthetic Bba_R0040 promoter adjacent to the target site. When expressing the bridge RNA in *cis*, pDonor encodes a full-length RE–LE sequence (298 bp), which naturally encodes the donor, the bridge RNA and a promoter to express the bridge RNA. When expressing the bridge RNA in *trans*, pDonor encodes a shortened donor sequence (22 bp) and a bridge RNA driven by the J23119 promoter and followed by the HDV ribozyme.

To measure excision, a Bba_R0040 promoter is separated from the GFP CDS by the donor site, 1 kb of intervening DNA sequence including an ECK120029600 to terminate transcription, and a target site on the same strand. Co-expression of a second plasmid encoding a bridge RNA and a T7-inducible IS621 recombinase results in the excision of the intervening 1-kb sequence, yielding GFP expression.

To measure inversion, a Bba_R0040 promoter is encoded adjacent to a top-strand donor sequence, followed by a GFP CDS and target sequence encoded on the bottom strand. Co-expression of a second plasmid encoding a bridge RNA and a T7-inducible IS621 recombinase results in the inversion of the GFP CDS (around 900 bp), yielding GFP expression.

In all GFP reporter assays, co-transformed cells were plated on fresh LB agar containing kanamycin, chloramphenicol and 0.07 mM IPTG to induce recombinase expression. Plates were incubated at 37 °C for 16 h and subsequently incubated at room temperature for 8 h. Hundreds of colonies were subsequently scraped from the plate, resuspended in TB and diluted to an appropriate concentration for flow cytometry. Around 50,000 cells were analysed on a Novocyte Quanteon Flow Cytometer to assess the fluorescence intensity of GFP-expressing cells. The mean fluorescence intensity of the population (including both GFP^+^ and GFP^−^ cells) is plotted as analysed with NovoExpress software (v.1.5.6). pRecombinant plasmids were isolated by picking GFP^+^ colonies under blue light, seeding in TB containing kanamycin and chloramphenicol, incubating for 16 h at 37 °C with shaking at 200 rpm, and isolating using the QIAprep Spin Miniprep kit. The isolated plasmids were sent for whole-plasmid sequencing to confirm recombination (Primordium Labs).

### Design of the oligo pool for systematic pairwise screening of bridge RNA target-binding loops and targets

A pooled screen was designed to test target and target-binding loop mismatch tolerance and relative efficiency across diverse guide sequences. Several categories of oligos were designed to answer different questions. First, 10,656 oligos were designed to test hundreds of different target guides with single-mismatch pairs. That is, for a given target, one position in the guide and the corresponding position in the target to generate all 4 × 4 = 16 combinations of nucleotides. Target guides were selected to reduce genomic off-targets. Next, 3,600 oligos were designed to test different combinations of double mismatches between target-binding loop and target. Next, 2,000 oligos were designed as an internal set of negative controls by ensuring that none of the 9 programmable positions (excluding the CT core) matched in the target-binding loop and the target. Next, another 1,800 oligos were designed to test more single-mismatch combinations, but did not include all 4 × 4 combinations in the target and the target-binding loop. Finally, 1,610 oligos were designed to test how mismatches in the dinucleotide core of the bridge RNA sequences affected the recombination efficiency. One unique barcode per amplicon was assigned at random, ensuring that no two barcodes were within two mismatches of each other. Each oligo encoded a synthetic Bba_R0040 promoter followed by a target sequence, a unique barcode, the J23119 promoter and the first 104 bases of the bridge RNA, which includes the 5′ stem-loop and target-binding loop. The oligos were ordered as a single pooled library from Twist Bioscience.

### Cloning of the oligo pool for systematic pairwise screening of bridge RNA target-binding loops and targets

A vector encoding the final 73 bp of the bridge RNA (the WT donor-binding loop) and a T7-inducible IS621 recombinase was digested using BsaI. The oligo library was amplified with primers encoding overhangs compatible with the digested vector for Gibson cloning. In brief, the library was cloned into the vector by Gibson cloning, and electroporated in Endura DUO electrocompetent cells (Biosearch Technologies). Hundreds of thousands of colonies were isolated for sufficient coverage of the oligo library, and plasmids containing library members were purified using the Nucleobond Xtra Midiprep kit (Macherey Nagel).

### Recombination assay with the library of bridge RNA target-binding loops and targets

The plasmid library encoding thousands of target and bridge RNA target-binding loop pairs was co-electroporated into E. cloni EXPRESS electrocompetent cells (Biosearch Technologies) along with a donor plasmid and an inactive kanamycin resistance gene. Recombination between the two plasmids results in the expression of the kanamycin resistance gene, allowing cell survival. After co-electroporation and recovery, cells were plated on bioassay dishes with LB agar. One plating condition, serving as the control, was LB agar with chloramphenicol and ampicillin, which maintain the plasmids but do not induce or require recombination. A second condition was LB agar with chloramphenicol, ampicillin, kanamycin and 0.1 mM IPTG; IPTG induces recombinase expression, prompting recombination, and kanamycin selects for cells that have induced recombination between the donor and the target plasmid. Both conditions were performed in two replicates. Recombination indicates a compatible target–target-binding loop pair within the library.

Hundreds of thousands of colonies were scraped from the bioassay dishes and had plasmid DNA extracted using the Nucleobond Xtra Midiprep kit (Macherey Nagel). After plasmid DNA isolation, samples were prepared for NGS. For DNA isolated from the control conditions, a PCR was used to amplify the barcodes specifying target and bridge RNA pairs to measure the distribution of barcodes without selecting conditions. For DNA isolated from selection conditions, a PCR was used to amplify the barcodes specifying target and bridge RNA pairs, with one primer priming from the donor plasmid and the other priming from the target plasmid such that only barcodes from recombinant plasmids were measured. The distribution of barcodes from recombinant plasmids was subsequently compared to the distribution of barcodes under control conditions.

### Analysis of target specificity screen

Amplicon sequences were processed using the bbduk tool^66^. Amplicon sequencing data were then aligned to their respective wild types using bwa-mem, with ambiguous nucleotides at all variable positions^67^. Barcodes were then extracted from the amplicons using custom Python scripts. Barcodes were mapped to the designed barcode library, tolerating single mismatches when making assignments. This resulted in a table of barcode counts per biological replicate. Using custom R scripts, the counts were normalized within each replicate using counts per million (CPM), which converts raw barcode counts into barcode counts per million barcodes. CPM values were then averaged across the two biological replicates in each condition. For the recombinant barcodes, CPM values were then corrected by the control barcode CPM values using a simple correction factor for each barcode, calculated by dividing the expected barcode CPM (assuming a uniform distribution) by the observed barcode CPM. These corrected CPM values were subsequently used in many of the individual analyses. Mismatch tolerance was assessed by limiting the analysis to the top quintile of the most efficient 4 × 4 single-mismatch sets, in which each set was ranked according to the barcode with maximum efficiency, and then averaging the percentage of total CPM within each set at each position. The motif of enriched nucleotides at each position was generated by determining the nucleotide composition of the top quintile of the most efficient target-binding loop–target pairs (without mismatches), and comparing this to the nucleotide composition of the entire set.

### IS621 genomic insertion assay with long-read sequencing

A plasmid was prepared that encoded a donor sequence adjacent to a constitutively expressed kanamycin resistance gene and a temperature-sensitive Rep101 protein. Plasmid replication of this donor plasmid was eliminated in cells upon growth at 37 °C, ensuring that cells encode a single copy of the donor plasmid. A cell line was prepared encoding this donor plasmid by transforming BL21(DE3) and making the resultant cell line chemically competent using the Mix & Go preparation kit (Zymo). The temperature-sensitive donor plasmid was then transformed with a second plasmid encoding a T7-inducible recombinase and a constitutively expressed bridge RNA. The donor-binding loop of the bridge RNA was programmed to recognize the donor sequence within the donor plasmid and the target-binding loop of the bridge RNA was programmed to recognize a target sequence in the BL21(DE3) *E. coli* genome. After transformation, cells were recovered and plated on 10-cm LB agar plates containing 0.02 mM IPTG, chloramphenicol and kanamycin; insertion of the donor plasmid and expression of the kanamycin resistance gene from the genome is required for cell survival. The thousands of resulting colonies, each with an insertion of the donor plasmid into the genome, were scraped from the plate. Genomic DNA was extracted from the pool of colonies using the Quick DNA Miniprep Plus kit (Zymo). Genomic DNA was then cleaned up using AMpure XP (Beckman Coulter) and sequenced using bacterial genome nanopore sequencing to at least 100× genome coverage.

Sequencing data were downsampled to a sequencing depth of 200× in reprogrammed bridge RNA experiments, and to a depth of 1,400× in the WT bridge RNA experiments. To identify long reads containing potential insertion junctions between the plasmid donor and the *E. coli* genome (NZ_CP053602.1), all individual reads were programmatically scanned for the presence of the terminal 20 nucleotides of the donor sequence, excluding the core. If a 20-bp sub-sequence of a read matched the 5′ terminus or 3′ terminus (allowing for up to two mismatches), then the read was split and the flanking sequences were written to separate files. These flanking sequences were then mapped back to the plasmid sequences and the *E. coli* genome using minimap2 (Li 2018), and assigned as originating from the plasmid or the *E. coli* genome according to whichever had the higher alignment score. Reads were then assigned to specific insertion junctions in the *E. coli* genome to identify precise insertion sites. Insertion sites that were within 5 bp of each other were merged together using bedtools merge^68^ and a representative insertion site was selected. For the reprogrammed bridge RNA genome insertion experiments, additional filters were applied to remove low-quality alignments and account for a low rate (<1%) of cross-sample contamination (possibly owing to index hopping). Low-quality predicted insertion sites were excluded only if they met certain criteria: either (1) occurring at a total insertion frequency of less than 1%; occurring at a Levenshtein distance of more than 2 nt from the 11-nt target and donor; and supported by a large fraction of clipped reads (more than 25%, indicating low alignment quality); or (2) occurring at a total insertion frequency of less than 1%; occurring at a Levenshtein distance of more than 2 nt from the 11-nt target and donor; and matching a high frequency (more than 1%) and close target match (Levenshtein distance of less than 3 nt) in a different sample (suggesting that index hopping across samples is likely). The total number of reads per site was subsequently used to determine the insertion specificity for each site.

Off-target sites were evaluated by calculating the Levenshtein distance between the 11-nt off-target and the 11-nt target and donor sequences. Sequences with a Levenshtein distance of more than 2 nt from the target and donor were further evaluated by searching for shared *k*-mer sequences in the 14-nt off-target, the 14-nt expected target and the 14-nt donor. To determine whether the off-target sequences were enriched for shared target or donor *k*-mers, the maximum-length shared *k*-mer distribution was generated and compared to a null distribution in which the 14-nt off-target sequences were randomly shuffled. This shuffling procedure was repeated 1,000 times to calculate the null distribution.

A computational pipeline was developed to identify potential structural variants (50 bp or greater in size) that were independent from the donor plasmid. All long-read nanopore sequences were aligned to the BL21(DE3) *E. coli* genome (NZ_CP053602.1) and the pDonor and pHelper plasmid sequences. Reads that aligned to the pDonor or pHelper sequences were then excluded from the *E. coli* genome alignment. These filtered alignments were analysed using fgsv v.0.0.1 (ref. ^69^). The tool geNomad was used to annotate a structural variant involving a possible prophage element^70^.

For the WT bridge RNA, REP elements were also identified and annotated to determine how frequently they were targeted. REP elements were identified by a BLAST search of three different known REP sequences collected from two different studies^11,16^. These query sequences were TGCCGGATGCGGCGTAAACGCCTTATCCGGCCTAC, GCCTGATGCGCTACGCTTATCAGGCCTACG and GCCTGATGCGACGCTGGCGCGTCTTATCAGGCCTACG.

### Design of the oligo pool for systematic screening of bridge RNA donor-binding loops and donors

A pooled screen was designed to test donor-binding loop programmability, mismatch tolerance and relative efficiency across diverse guide sequences. Several categories of oligos were designed to answer different questions. Donor sequences were selected to reduce predicted genomic off-targets. First, 13,593 oligos were designed that included complete single-mismatch scans across 100 distinct donors, including all position 4 × 4 = 16 mismatches with the donor at the corresponding position. Next, 5,000 completely random donor guides were selected and paired with a perfectly matching donor for the analysis of a high number of diverse donor sequences. Finally, 2,297 oligos to test single-mismatch and double-mismatch scans of the WT donor sequence and 4 other functional donors were included. Next, 50 negative control oligos were included that ensured that none of the 9 programmable positions (excluding the CT core) matched in the donor-binding loop and donor. Each oligo encoded a partial sequence of the IS621 RE (52 bp 5′ of the CT core), the reprogrammed donor sequence and a full-length LE (191 bp) encoding a bridge RNA as found in the WT system, such that expression of the bridge RNA would be mediated by the natural promoter in *cis*. The donor site sequence and donor-binding loop sequence of the bridge RNA were modified in each member according to the description above, whereas the target-binding loop of the bridge RNA was constant and programmed to recognize the target sequence T5, which is orthogonal to the BL21(DE3) *E. coli* genome. The oligo was flanked on both ends with sequences suitable for Golden Gate cloning into a desired plasmid backbone. All oligos were ordered as a single pooled library from Twist.

### Cloning of the oligo pool for screening of bridge RNA donor-binding loops and donors

First, a vector was constructed encoding a kanamycin resistance gene with no promoter on the bottom strand, followed by the first 61 bp of the IS621 RE sequence. This was followed by a BsaI landing pad site for Golden Gate cloning, an HDV ribozyme sequence and a unique molecular identifier (UMI) of length 12. The UMI backbone was pre-digested by BsaI and the oligo library was cloned into the backbone through Golden Gate cloning after amplification with appropriate primers, such that the full-length IS621 RE was reconstituted and the LE containing the bridge RNA was directly adjacent to the HDV ribozyme sequence. The resulting library was electroporated in Endura DUO electrocompetent cells (Biosearch Technologies). Hundreds of thousands of colonies were isolated for sufficient coverage of the oligo library, and plasmids containing library members were purified using the Nucleobond Xtra Midiprep kit (Macherey Nagel).

### Recombination assay with the library of bridge RNA donor-binding loops and donors

The plasmid library encoding thousands of donor and bridge RNA donor-binding loop pairs was co-electroporated into E. cloni EXPRESS electrocompetent cells (Biosearch Technologies) with a target plasmid encoding the T5 target sequence and a T7-inducible IS621 recombinase. Recombination between the two plasmids results in the expression of the kanamycin resistance gene, allowing cell survival. After co-electroporation and recovery, cells were plated on bioassay dishes with LB agar. One plating condition, serving as the control, was LB agar with chloramphenicol and ampicillin, which maintain the plasmids but do not induce or require recombination. A second condition was LB agar with chloramphenicol, ampicillin, kanamycin and 0.07 mM IPTG; IPTG induces recombinase expression, prompting recombination, and kanamycin selects for cells that have induced recombination between the donor and the target plasmid. Both conditions were performed in two replicates. Recombination indicates a compatible target–target-binding loop pair within the library.

Hundreds of thousands of colonies were scraped from the bioassay dishes and had plasmid DNA extracted using the Nucleobond Xtra Midiprep kit (Macherey Nagel). After the isolation of plasmid DNA, samples were prepared for NGS. For DNA isolated from the control conditions, a PCR was used to amplify the UMI specifying donor and bridge RNA pairs to measure the distribution of UMIs without selecting conditions. For DNA isolated from selection conditions, a PCR was used to amplify the UMIs specifying donor and bridge RNA pairs, with one primer priming from the donor plasmid and the other priming from the target plasmid such that only UMIs from recombinant plasmids were measured. The distribution of UMIs from recombinant plasmids was subsequently compared to the distribution of UMIs under control conditions. UMIs were initially mapped to donor–bridge RNA pairs by amplifying a region of the input donor library such that information about all variable sites within the full length of the RE–LE was captured in addition to the adjacent UMI.

### Analysis of the donor specificity screen

All amplicon sequence data were preprocessed using bbduk to remove adapters. Next, UMIs were mapped to their respective oligos. This was done by aligning to the expected amplicon sequence with ambiguous N nucleotides in all of the variable positions using bwa-mem^67^. UMIs were then determined from the alignments, and combined with the variable LDG and RDG to guarantee the uniqueness of each UMI to each oligo. Next, control and recombinant samples were analysed in much the same way as the previously described target screen, but UMIs were counted rather than assigned barcodes. Next, UMI counts were converted to CPM, averaged across two biological replicates and normalized according to the correction factors calculated in the control condition. These CPM values were then analysed across different oligo categories to assess mismatch tolerance, how distance from the wild-type donor affects efficiency and which nucleotide sequences were favoured or disfavoured at each position in the donor.

### Additional analyses of natural IS110 sequences

Natural IS621 target sites were extracted from the genomic sequence database by searching for exact matches to the 1,277-bp IS621, excluding the core. These target sequences were then clustered using mmseqs2 and the parameters ‘easy-cluster --cov-mode 0 -c 0.800 --min-seq-id 0.800’^52^. This search and clustering identified 272 distinct target sites, which were then analysed to identify a conserved target motif and compared with the experimental observed IS621 target sequences in the *E. coli* BL21(DE3) genome.

A paired alignment of target sites and bridge RNA sequences was analysed to determine how the target site motif changed as the guide RNAs were varied. All aligned bridge RNA sequences that lacked gaps in the nine-base LTG and the four-base RTG were first identified. Next, only LTG and RTG sequences with CT core guides were selected. Next, only target-binding loops with more than 20 associated target sites were kept. For each of these unique remaining target-binding loops, a consensus sequence of the motif was constructed by selecting the most common nucleotide at each of the 11 target positions. If there were ties, then the position was represented by the ambiguous IUPAC character N. These consensus target sites were then compared with the expected target sites to determine how closely they matched.

### Reporting summary

Further information on research design is available in the Nature Portfolio Reporting Summary linked to this article.

## Online content

Any methods, additional references, Nature Portfolio reporting summaries, source data, extended data, supplementary information, acknowledgements, peer review information; details of author contributions and competing interests; and statements of data and code availability are available at 10.1038/s41586-024-07552-4.

### Supplementary information

Supplementary Information This file contains Supplementary Figures 1–5 and Supplementary Note 1.

Reporting Summary

Supplementary Tables Supplementary Tables 1–5.

Supplementary Data 1 Input sequences and covariation scores from Figure 2b and Extended Data Figure 10a.

Supplementary Data 2 Input sequences for analyses performed in Extended Data Figure 5a, 5d, and 5e.

Additional Code 1

Additional Code 2

## Data Availability

The NGS dataset is available on the NCBI Sequence Read Archive at Bioproject PRJNA1013328. Reference IS110 sequences and metadata were accessed through the ISfinder website (https://isfinder.biotoul.fr/). Additional genomic and metagenomic sequences were analysed to identify IS110 elements, and these sequences were acquired from public databases including NCBI (https://www.ncbi.nlm.nih.gov/), UHGG/MGnify (https://www.ebi.ac.uk/metagenomics), JGI IMG (https://img.jgi.doe.gov/), the Gut Phage Database (https://ftp.ebi.ac.uk/pub/databases/metagenomics/genome_sets/gut_phage_database/), the Human Gastrointestinal Bacteria Genome Collection (http://ftp.ebi.ac.uk/pub/databases/metagenomics/), Youngblut et al. animal gut metagenomes (http://ftp.tue.mpg.de/ebio/projects/animal_gut_metagenome_assembly/), MG-RAST (https://www.mg-rast.org/) and Tara Oceans samples (https://ocean-microbiome.embl.de/companion.html).

## Code availability

Code accompanying this study is available at https://github.com/hsulab-arc/BridgeRNA2024. A Python package for designing bridge RNA sequences is available at https://github.com/hsulab-arc/BridgeRNADesigner. This design tool is also available through a web application at https://www.arcinstitute.org/tools/bridge.
