# Supplementary material for: Bridge RNAs direct programmable recombination of target and donor DNA
Source: Nature. 2024 Jun 26;630(8018):984–93. doi: 10.1038/s41586-024-07552-4 (PMC11208160; doi:10.1038/s41586-024-07552-4)
Supplement: Supplementary file 2 — Reporting Summary [file 41586_2024_7552_MOESM2_ESM.pdf]

nature portfolio

Corresponding author(s): Patrick D. Hsu

Last updated by author(s): Mar 29, 2024

Reporting Summary

Nature Portfolio wishes to improve the reproducibility of the work that we publish. This form provides structure for consistency and transparency in reporting. For further information on Nature Portfolio policies, see our Editorial Policies and the Editorial Policy Checklist.

Statistics

For all statistical analyses, confirm that the following items are present in the figure legend, table legend, main text, or Methods section.

n/a	Confirmed	
[ ]	[x]	The exact sample size (*n*) for each experimental group/condition, given as a discrete number and unit of measurement
[x]	[ ]	A statement on whether measurements were taken from distinct samples or whether the same sample was measured repeatedly
[x]	[ ]	The statistical test(s) used AND whether they are one- or two-sided<br>*Only common tests should be described solely by name; describe more complex techniques in the Methods section.*
[x]	[ ]	A description of all covariates tested
[x]	[ ]	A description of any assumptions or corrections, such as tests of normality and adjustment for multiple comparisons
[ ]	[x]	A full description of the statistical parameters including central tendency (e.g. means) or other basic estimates (e.g. regression coefficient) AND variation (e.g. standard deviation) or associated estimates of uncertainty (e.g. confidence intervals)
[x]	[ ]	For null hypothesis testing, the test statistic (e.g. *F*, *t*, *r*) with confidence intervals, effect sizes, degrees of freedom and *P* value noted<br>*Give P values as exact values whenever suitable.*
[x]	[ ]	For Bayesian analysis, information on the choice of priors and Markov chain Monte Carlo settings
[x]	[ ]	For hierarchical and complex designs, identification of the appropriate level for tests and full reporting of outcomes
[x]	[ ]	Estimates of effect sizes (e.g. Cohen's *d*, Pearson's *r*), indicating how they were calculated

Our web collection on statistics for biologists contains articles on many of the points above.

Software and code

Policy information about availability of computer code

Data collection	Standard instrument software was used for data collection (see Methods). Agilent Quanteon NovoCyte was used for flow cytometry data collection using NovoExpress software (Version 1.5.6). Microscale thermophoresis (MST) was carried out using a Monolith NT.115pico Series instrument (NanoTemper technologies). Data were analyzed using the NanoTemper MO.affinity analysis (v3.0.5) software package and raw data were plotted on GraphPad Prism (10.2.0) for visualization.
Data analysis	Prodigal v2.6.3, MMseqs2 v13.45111, HMM ER v3.lb2, ggseqlogo v0.l, ggtree v3.0.2, prokka vl.14.5, iqtree2 v2.l.4-beta, mafft v7.310, MGEfinder vl.0.6, BLAST v2.12.0+, ConsAlifold v0.l.17, INFERNAL vl.1.4, CCMpred v0.3.2, BBTools v39.01, BWA v0.7.17-r1188, minimap2 v2.22-r1101, bedtools v2.30.0, fgsv v0.0.1. Custom code is available at https://github.com/hsulab-arc/BridgeRNA2024 and https://github.com/hsulab-arc/BridgeRNADesigner.

For manuscripts utilizing custom algorithms or software that are central to the research but not yet described in published literature, software must be made available to editors and reviewers. We strongly encourage code deposition in a community repository (e.g. GitHub). See the Nature Portfolio guidelines for submitting code & software for further information.

Data

Policy information about availability of data

All manuscripts must include a data availability statement. This statement should provide the following information, where applicable:

- Accession codes, unique identifiers, or web links for publicly available datasets
- A description of any restrictions on data availability
- For clinical datasets or third party data, please ensure that the statement adheres to our policy

The next-generation sequencing dataset is available on the NCBI SRA at Bioproject PRJNA1013328. Reference IS110 sequences and metadata were accessed through the ISfinder website (https://isfinder.biotoul.fr/). Additional genomic and metagenomic sequences were analyzed to identify IS110 elements, and these sequences were acquired from public databases including NCBI (https://www.ncbi.nlm.nih.gov/), UHGG/MGnify (https://www.ebi.ac.uk/metagenomics), JGI IMG (https://img.jgi.doe.gov/), the Gut Phage Database (https://ftp.ebi.ac.uk/pub/databases/metagenomics/genome_sets/gut_phage_database/), the Human Gastrointestinal Bacteria Genome Collection (http://ftp.ebi.ac.uk/pub/databases/metagenomics/), Youngblut et al. animal gut metagenomes (http://ftp.tue.mpg.de/ebio/projects/animal_gut_metagenome_assembly/), MG-RAST (https://www.mg-rast.org/), and Tara Oceans samples (https://ocean-microbiome.embl.de/companion.html).

Research involving human participants, their data, or biological material

Policy information about studies with human participants or human data. See also policy information about sex, gender (identity/presentation), and sexual orientation and race, ethnicity and racism.

Reporting on sex and gender	N/A
Reporting on race, ethnicity, or other socially relevant groupings	N/A
Population characteristics	N/A
Recruitment	N/A
Ethics oversight	N/A

Note that full information on the approval of the study protocol must also be provided in the manuscript.

Field-specific reporting

Please select the one below that is the best fit for your research. If you are not sure, read the appropriate sections before making your selection.

☒ Life sciences ☐ Behavioural & social sciences ☐ Ecological, evolutionary & environmental sciences

For a reference copy of the document with all sections, see nature.com/documents/nr-reporting-summary-flat.pdf

Life sciences study design

All studies must disclose on these points even when the disclosure is negative.

Sample size	Sample sizes of n=3 were chosen for experiments in cells, as is standard. The exception to this were experiments requiring high cost next generation sequencing, including high throughput screens and genome insertion experiments, where n=2 was performed.
Data exclusions	No data was excluded.
Replication	All attempts at replication were successful.
Randomization	Randomization was determined not necessary for working with bacterial cell lines.
Blinding	Blinding was determined not necessary for this non-clinical research.

Reporting for specific materials, systems and methods

We require information from authors about some types of materials, experimental systems and methods used in many studies. Here, indicate whether each material, system or method listed is relevant to your study. If you are not sure if a list item applies to your research, read the appropriate section before selecting a response.

Materials & experimental systems

n/a	Involved in the study
☒	☐ Antibodies
☒	☐ Eukaryotic cell lines
☒	☐ Palaeontology and archaeology
☒	☐ Animals and other organisms
☒	☐ Clinical data
☒	☐ Dual use research of concern
☒	☐ Plants

Methods

n/a	Involved in the study
☒	☐ ChIP-seq
☐	☒ Flow cytometry
☒	☐ MRI-based neuroimaging

Plants

Seed stocks: N/A

Novel plant genotypes: N/A

Authentication: N/A

Flow Cytometry

Plots

Confirm that:

☒ The axis labels state the marker and fluorochrome used (e.g. CD4-FITC).

☒ The axis scales are clearly visible. Include numbers along axes only for bottom left plot of group (a 'group' is an analysis of identical markers).

☒ All plots are contour plots with outliers or pseudocolor plots.

☒ A numerical value for number of cells or percentage (with statistics) is provided.

Methodology

Sample preparation: All cells were E. coli. Cells were scraped from agar plates, resuspended in DPBS, and diluted to the appropriate concentration.

Instrument: Agilent NovoCyte Quanteon

Software: NovoExpress software (Version 1.5.6) was used for flow cytometry analysis.

Cell population abundance: At least 50,000 cells were analyzed.

Gating strategy: The majority (95%) of cells were gated on FSC/SSC. No further gating was performed.

☒ Tick this box to confirm that a figure exemplifying the gating strategy is provided in the Supplementary Information.
